# Molecular and Biomechanical Adaptations to Mechanical Stretch in Cultured Myotubes

**DOI:** 10.3389/fphys.2021.689492

**Published:** 2021-08-02

**Authors:** Dapeng Ren, Jing Song, Ran Liu, Xuemin Zeng, Xiao Yan, Qiang Zhang, Xiao Yuan

**Affiliations:** ^1^Department of Stomatology Medical Center, The Affiliated Hospital of Qingdao University, Qingdao, China; ^2^College of Dentistry, Qingdao University, Qingdao, China

**Keywords:** myotube, mechanical stretch, biomechanical adaptation, mechanoresponse, skeletal muscle

## Abstract

Myotubes are mature muscle cells that form the basic structural element of skeletal muscle. When stretching skeletal muscles, myotubes are subjected to passive tension as well. This lead to alterations in myotube cytophysiology, which could be related with muscular biomechanics. During the past decades, much progresses have been made in exploring biomechanical properties of myotubes *in vitro*. In this review, we integrated the studies focusing on cultured myotubes being mechanically stretched, and classified these studies into several categories: amino acid and glucose uptake, protein turnover, myotube hypertrophy and atrophy, maturation, alignment, secretion of cytokines, cytoskeleton adaption, myotube damage, ion channel activation, and oxidative stress in myotubes. These biomechanical adaptions do not occur independently, but interconnect with each other as part of the systematic mechanoresponse of myotubes. The purpose of this review is to broaden our comprehensions of stretch-induced muscular alterations in cellular and molecular scales, and to point out future challenges and directions in investigating myotube biomechanical manifestations.

## Background

Human body has over 600 skeletal muscles that are involved in locomotion, mastication, maintaining posture. Being an elastic and flexible tissue, skeletal muscles undergo passive lengthening under various conditions, and rapidly adapt to these stretching environments (e.g., daily growth and elongation of bone, stretching rehabilitation training, distraction osteogenesis, and functional orthopedic therapy). On organ and tissue scales, extensive studies have been made to passively stretch skeletal muscles *in vitro*, and to evaluate the resultant biomechanical adaptions of muscles to stretching (see review Mohammadkhah et al., 2016).

Anatomically, muscles comprised mainly of muscle fibers with long cylindrical, multinucleated structures, which are called myotubes. Myotubes have been credited with morphological, metabolic, and biochemical properties similar to those of mature muscle fibers (Berggren et al., 2007; Aas et al., 2013), thus rendering myotubes a widely used model to study the metabolism and biochemistry of skeletal muscles on the cellular, subcellular and molecular scales. Over the past decades, numerous *in vitro* studies reported the molecular and biomechanical alterations of cultured myotubes in response to mechanical stretch. However, there is no comprehensive review regarding these various biomechanical responses of myotubes.

In this review, we integrated distinct mechanoresponses of myotubes under various stretching conditions, which included the following aspects: amino acid and glucose uptake, protein turnover, hypertrophy and atrophy, maturation, alignment, ion channel activation, secretion of cytokines, cytoskeletal adaption, damage, and oxidative stress. We also addressed the interconnections among different studies, and discussed the potential molecular mechanisms. Finally, future challenges and directions were proposed in exploring biomechanical adaptations to mechanical stretch in cultured myotubes.

## Amino Acid Uptake

Amino acid uptake and incorporation are primary steps for muscle protein synthesis. In 1979, Vandenburgh et al. first established an *in vitro* stretching system that applied static stretch to myotubes of chicken breast muscles (Vandenburgh and Karlisch, 1989). By measuring α-amino isobutyric acid (AIB), they found that amino acid uptake by myotubes was increased with stretching magnitude from 7.5 to 13%, but decreased under 20.8% stretch (Vandenburgh and Kaufman, 1979, 1981). The amino acid uptake in myotubes was not elevated until 30 min stretching stimulation, and remained at high level for 2–6 h (Vandenburgh and Kaufman, 1981).

Amino acid transport into stretched myotubes was dependent on activated Na ^+^ pump (Vandenburgh and Kaufman, 1981). Consistently, drugs like tetrodotoxin and ouabain which inhibit Na^+^ influx blocked AIB accumulation in stretched myotubes (Vandenburgh and Kaufman, 1981; Vandenburgh, 1982). Moreover, depolarizing myotube's membrane potential by increasing the extracellular potassium level also reduced the stretch-induced AIB uptake (Vandenburgh and Kaufman, 1982). Altogether, these data highlighted the importance of Na^+^ pump and membrane potential of myotubes in uptaking amino acid during stretching stimuli.

Another intriguing finding was that while stretching stimuli could promote amino acid transport into myotubes, addition of conditioned media from stretched myotubes had no effect on amino acid transportation in unstretched myotubes (Vandenburgh, 1983). Therefore, elevated amino acid uptake into stretched myotubes did not result from some secreted factors released during stretching, but seemed to be attributed to the direct influences of stretch on myotubes (Vandenburgh, 1983).

The L-type amino acid transporter 1 (LAT1), which plays major roles in cellular amino acid uptake, is expressed in skeletal muscle tissue in both humans and rats (Drummond et al., 2010; Murakami and Yoshinaga, 2013). Nakai et al. (2018) found that 15% uniaxial stretch prompted LAT1 protein and mRNA expressions in C2C12 myotubes. However, whether stretch-elevated LAT1 contributed to amino acid uptake was not explored in their study, which would be an interesting problem to be addressed in future.

## Protein Synthesis and Degradation

It is well-known that proteins in animal cells are being synthesized and degraded continuously. Mechanical stretch could promote protein synthesis of myotubes (Vandenburgh et al., 1989, 1990; Perrone et al., 1995; Clarke and Feeback, 1996). Growth factors like insulin-like growth factor 1 (IGF-1) and fibroblast growth factor (FGF) were released from myotubes by stretch, which facilitated myotube protein synthesis via autocrine mode of action (Perrone et al., 1995; Clarke and Feeback, 1996). In spite of the elevated protein synthesis in myotubes under mechanical stretch, the stretching parameters (frequency, intensity, duration) being applied is still worthy of attention. For example, when C2C12 myotubes were subjected to cyclic strain at strain rates ranging from 1.4 to 70% s^−1^, protein synthesis increased at strain rates up to 25% s^−1^ and then decreased at higher strain rates (Clark et al., 2001). In addition, protein synthesis in myotubes is optimally stimulated when the stretching intensity is 8% (Vandenburgh et al., 1989). Furthermore, short-term stretch and long-term stretch might facilitate myotube protein synthesis via different mechanisms, as the later required serum in the culture medium while the former did not (Vandenburgh et al., 1989). These studies possibly reflected the complex regulation of protein synthesis by a diversity of signaling pathways that are differently activated in myotubes subjected to various stretching conditions.

Stretching stimuli was also reported to have bidirectional regulation of protein degradation in myotubes. For example, studies by Vandenburgh et al. (1989, 1990) displayed increased protein degradation rate in myotubes under intermittent stretch, which was attributed to elevated activities of proteinases, such as calpain, cathepsin H, and metalloproteinase-7 (MMP-7). Conversely, their another study proved that static stretch profoundly inhibited the degradation of long lived proteins in myotubes (Vandenburgh and Kaufman, 1980). We assumed that the discrepancy between these studies could be ascribed to the distinct stretching patterns (see Table 1 for detailed information).

**Table 1:** The effects of culturing conditions and stretching regimes on protein turnover, hypertrophy, and atrophy of myotubes.

**Cells**		**Stretching parameters**				**Major effects on protein turnover and myotube hypertrophy**	**References**
**Culture before stretch**	**Culture during stretch**	**Stretching mode**	**Amplitude**	**Frequency**	**Regimen**		
Avian primary myoblasts were fused to form myotubes in medium containing 10% horse serum and 5% chicken embryo extract for 72 h	Culture in medium containing 10% horse serum and 5% chicken embryo extract	Radial; static; continuous	10.80%	NA	8 and 18 h	Inhibited protein degradation	Vandenburgh and Kaufman, 1980
Avian primary myoblasts were fused to form myotubes in medium containing 10% horse serum and 5% chicken embryo extract for 48 h, and were embedded in collagen gel matrix for several days	Culture in medium containing 10% horse serum and 5% chicken embryo extract	Radial; cyclic; intermittent	20%	0.25 HZ	Stretch and relax during a 20 s period followed by a 10 s rest; this pattern is repeated three times, followed by a 30-min rest period. The entire stretching period ranges from hours to days	Inhibited protein synthesis during short-term stretch; elevated protein synthesis and myotube diameter during long-term stretch	Vandenburgh et al., 1989, 1990
Avian primary myoblasts were fused to form myotubes in medium containing 10% horse serum and 5% chicken embryo extract for 48 h, and were embedded in collagen gel matrix for several days	Culture in serum-free medium containing basal medium and bovine serum albumin, as well as different levels of exogenous IGF-1 or insulin	Radial; cyclic; intermittent	12%	0.25 HZ	Stretch and relax during a 20 s period followed by a 10 s rest; this pattern is repeated three times, followed by a 30-min rest period. The entire stretching period ranges from hours to days	Elevated protein synthesis during short-term stretch	Perrone et al., 1995
Human skeletal muscle cells (HSkMC) were fused to form myotubes in F12/DMEM medium containing 5% horse serum	Culture in serum-free medium	Radial; cyclic; intermittent	10 and 20%	0.25 HZ	Stretch and relax during 20 s followed by a 30 s rest period, this pattern is repeated five times, followed by a 30-min rest period. This cycle continued for a 4 h period followed by a 12 h recovery period. The entire stretching period lasts for 7 days	Elevated protein synthesis	Clarke and Feeback, 1996
C2C12 myoblasts were fused to form myotubes in serum-free medium	Culture in DMEM containing 10% FBS	Radial; cyclic; intermittent	6.70%	0.25 HZ	Stretch and relax during a 20 s period followed by a 10 s rest; this pattern is repeated three times, followed by a 30-min rest period. The entire stretching period lasts for 48 h	Elevated protein synthesis	Frenette and Tidball, 1998
C2C12 myoblasts were fused to form myotubes in medium containing 10% horse serum and 1% chicken embryo extract, co-cultured with 4% C3H10T fibroblasts	Culture in serum-free medium	Uniaxial; cyclic; continuous	7%	Strain rates ranges from 1.4% per second to 70% per second (0.1 −5 HZ)	1 h	Significantly elevated protein synthesis at rate of 14% per second (1 HZ), inhibited protein synthesis at rate >30%	Clark et al., 2001
C2C12 myoblasts were fused to form myotubes in medium containing 5% horse serum	Culture in serum-free medium containing bovine serum albumin (BSA)	Uniaxial; cyclic; continuous	10%	1/6 HZ	3 days	Elevated myotube diameter	Adachi et al., 2003
C2C12 myoblasts were fused to form myotubes in medium containing 2% horse serum	Culture in medium containing 2% horse serum	Radial; cyclic; intermittent	12%	0.7 HZ	Stretched 1 h/d for 5 days (5dSTR) or for 2 days followed by 3 days cessation of stretch (2dSTR3dCES)	Elevated myotube diameter during 5dSTR and inhibited myotube diameter during 2dSTR3dCES	Soltow et al., 2013
C2C12 myoblasts were fused to form myotubes in medium containing 2% horse serum supplemented with insulin	Culture in medium containing 2% horse serum	Radial; cyclic; continuous	15%	1 HZ	6 h	Elevated myotube diameter immediately after stretch and returned to control level after cessation of stretch	Ilaiwy et al., 2016
C2C12 myoblasts were fused to form myotubes in medium containing 2% horse serum	Culture in medium containing 2% horse serum	Uniaxial; cyclic; continuous	(a) 15% (b) 10% (c) 10% (d) 2% (e) 2%	(a) 1 HZ (b) 1 HZ (c) 0.25 HZ (d) 0.25 HZ (e) 0.25 HZ	(a) 15 min (b) 1 h (c) 1 h (d) 12 h (e) 24 h	Increased anabolism at low strain, low frequency for long duration; increased catabolism at high strain, high frequency for intermediate or short duration;	Moustogiannis et al., 2020
L6 myoblasts were fused to form myotubes in medium containing 2% horse serum	Culture in medium containing 2% horse serum	Radial; cyclic; continuous	18%	1/6 HZ	96 h	Elevated protein synthesis	Yamashita-Goto et al., 2002; Goto et al., 2003
C2C12 myoblasts were fused to form 3D cultured myotubes in medium containing 2% horse serum	Culture in medium containing 2% horse serum	Uniaxial; static; continuous	15%	NA	Continuously increasing load to achieve 15% stretch over 1 h; maintain static 15% stretch for further 2 h	Elevated myotube diameter 21 and 45 h after stretch	Aguilar-Agon et al., 2019
C2C12 myoblasts were fused to form myotubes in medium containing 2% horse serum	Culture in tumor cell conditioned medium (CM) was diluted to 20% in medium containing 2% horse serum	Radial; cyclic; continuous	6%	0.5 HZ	2 daily series of 2 h cyclic stretching, with a 3 h-pause between them	CM culture led to decreased diameter of myotubes and reduced fusion index, which was counteracted by stretching	Baccam et al., 2019
C2C12 myoblasts were fused to form myotubes in medium containing 1% heat-inactivated FBS	Culture in medium containing 2% horse serum and 0.2 μM doxorubicin (DOX)	Radial; static; continuous	5%	NA	Stretch was started 30 min following the first DOX treatment, lasted for 1 h, and media were replaced with fresh media with or without DOX for a duration of 23 h	DOX inhibited protein synthesis and promoted protein degradation, which was counteracted by stretching	Guigni et al., 2019
Avian primary myoblasts were fused to form myotubes in medium containing 10% horse serum and 5% chicken embryo extract for 96–120 h	Serum-free medium containing varying concentrations of dexamethasone (Dex)	Radial; cyclic; continuous	10%	0.25 HZ	Stretch and relax during a 20 s period followed by a 10 s rest; this pattern is repeated three times, followed by a 5-min rest period. Thus, the cells were mechanically stimulated for a total of 60 s every 6 min 20 s	Dex inhibited protein synthesis, which was counteracted by stretching	Chromiak and Vandenburgh, 1992, 1994
C2C12 myoblasts were fused to form 3D cultured myotubes in medium containing 2% horse serum	Culture in medium containing 2% horse serum	Uniaxial; static; continuous	15%	NA	Continuously increasing load to achieve 15% stretch over 1 h; maintain static 15% stretch for further 2 h	Dex induced myotube atrophy with decreased diameter, which was counteracted by stretching	Aguilar-Agon et al., 2020

Prostaglandins (PG) are well-known regulators of protein turnover in skeletal muscle. Specifically, prostaglandin E2 (PGE2) and prostaglandin F2α (PGF2α) mediate muscle protein degradation and protein synthesis, respectively. Moreover, the productions of both prostaglandins correlated well with protein degradation and synthesis in stretched myotubes, and inhibition of prostaglandin efflux by indomethacin partially blocked both stretch-induced protein degradation and synthesis (Vandenburgh et al., 1990).

**Figure 1:**
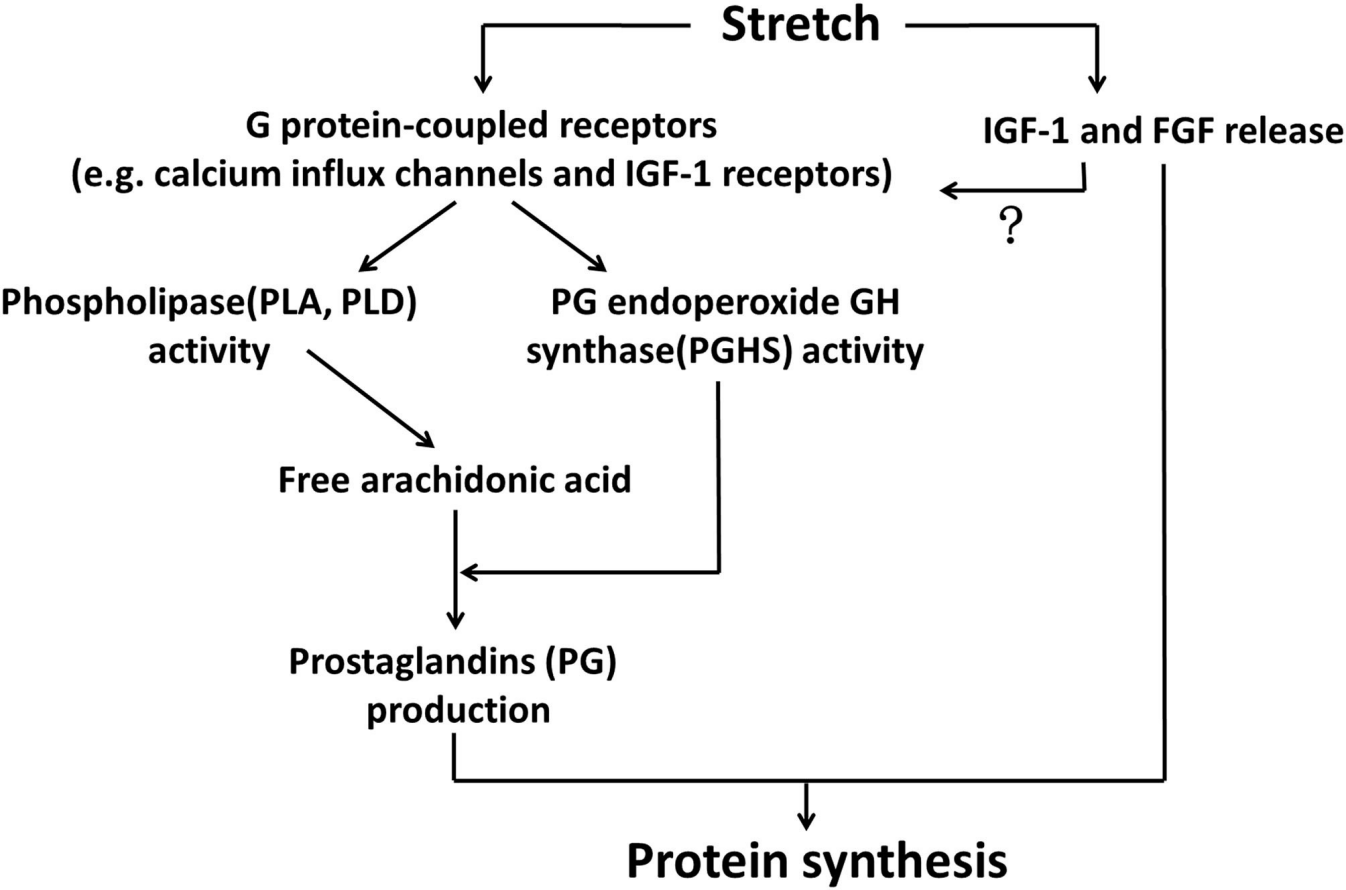
Stretch-released growth factors and prostaglandins regulated protein synthesis of myotubes. Stretch activated G protein-coupled receptors, such as calcium influx channels and IGF-1 receptors, in the membrane of myotubes, leading to elevated activities of phospholipases (PLA, PLD) and PG endoperoxide GH synthase (PGHS). PLA and PLD liberated arachidonic acid, which was subsequently converted by PGHS into prostaglandins production, such as PGE2 and PGF2. On the other hand, stretching stimuli could also promote growth factors (IGF1 and FGF) that participated in myotube protein synthesis through binding to their specific receptors. Whether these two major mechanisms acted simultaneously to regulate anabolism of myotubes under stretching environment would be a matter of interest.

Regarding the mechanisms through which mechanical stretch stimulated PG production and secretion from myotubes, Vandenburgh et al. (1993, 1995) established a relatively complete pathway consisting of two main regulatory steps. Firstly, stretching stimuli activated phospholipase A (PLA) and phospholipase D (PLD), leading to the liberation of arachidonic acid. Secondly, the activity of PG endoperoxide GH synthase (PGHS, also known as cyclooxygenase, COX) was elevated in myotubes under mechanical stretching as well, which converts arachidonic acid into PG production. The potential roles of PLA, PLD and PGHS in regulating myotube protein synthesis during mechanical tension were also confirmed by the prohibitory effects of their inhibitors on protein synthesis (Vandenburgh et al., 1993, 1995). With respect to the upstream events of stretch-induced activation of PLA, PLD and PGHS, stretch-sensitive G protein-coupled receptors such as calcium channels and IGF-1 receptors were possibly involved, as the inhibitor of G proteins partially blocked activities of PLD and PGHS, production of PG, as well as protein synthesis in stretched myotubes (Vandenburgh et al., 1995; Figure 1).

Another group of proteins involving in protein turnover is heat shock protein (HSP) that is normally activated by heat stress. Yamashita-Goto et al. (2002) and Goto et al. (2003) discovered that protein levels of HSP72 and HSP90 were significantly elevated in stretched myotubes, accompanied by accelerated protein synthesis. Therefore, the authors assumed that positive correlation between protein synthesis and expressions of these HSPs might be suggestive of their implication in stretch-induced myotube protein synthesis.

## Myotube Hypertrophy and Atrophy

The growth of skeletal muscle has two major forms: hyperplasia and hypertrophy. The former results from increased muscle cell proliferation, while the latter refers to increased diameter of muscle fibers that requires new myofibrillar protein synthesized in myotubes. It was reported that 2–4 days of mechanical tension significantly increased mean myotube diameter from 19 to 22 μm (Vandenburgh et al., 1989). Notably, stretch-induced myotube hypertrophy was dependent upon serum in culture medium. Stretch could only prevent myotube atrophy but not stimulate myotube hypertrophy in basal medium without serum (Vandenburgh et al., 1989). Consistently, Adachi et al. (2003) observed that stretching-promoted hypertrophy was accompanied by uptake of albumin, which is present in serum and cannot be synthesized in muscle cells. The physiological implication of the transport of albumin into stretched myotubes was assumed to modulate the Ca^2+^ pump of the sarcoplasmic reticulum (Adachi et al., 2003).

To best simulate the *in vivo* muscular stretching environment, Lewis et al. developed a 3D cultured-myotube stretching system by seeding myotubes within extracellular matrix (ECM) that was subjected to uniaxial tension. Their studies demonstrated that mechanical stretching of 3D cultured-myotubes led to raised expressions of hypertrophy-related genes such as matrix metalloprotease-2 and 9 (MMP-2,9), IGF-1, Insulin-like growth factor binding protein-2 and 5 (IGFBP-2,5) (Player et al., 2014; Aguilar-Agon et al., 2019). Moreover, average myotube width was also higher in 3D stretched myotubes than non-stretched 3D cultures (Aguilar-Agon et al., 2019). This *in vitro* model of mechanically stretching 3D cultured-myotube could greatly mimic exercise-induced hypertrophy of skeletal muscles.

In contrast to myotube hypertrophy under consecutive mechanical stimulation, cessation of stretch induced atrophy of myotubes with diminished diameter (Ilaiwy et al., 2016). This was attributed to activation of atrophy signaling, which resulted in proteolysis of αII-spectrin and talin (Soltow et al., 2013). Interestingly, intensive stretch with higher strain and frequency along with shorter duration also promoted expressions of atrophy gene atrogin-1 and muscle ringer finger-1 (MuRF1) in myotubes, in comparison with myotube growth and hypertrophy by stretching at lower strain and frequency for longer duration (Moustogiannis et al., 2020). Thus, both stretching cessation and overstretching could cause *in vitro* myotube atrophy, reflecting the detrimental effect of both muscle disuse and overuse on muscular metabolism.

Apart from muscular disuse or overuse, both cancer and its treatment have profound effect on skeletal muscle atrophy, which could be partially ameliorated by physical exercises. Consistently, Baccam et al. (2019) reported that tumor-derived factor activin induced myotube atrophy *in vitro*. Application of mechanical stress was sufficient to rescue myotubes from activin-induced myofiber atrophy, through the secretion of follistatin that could compete with activin for binding to activin type II B receptor (ActRIIB). On the other hand, chemotherapeutic doxorubicin (DOX), one ordinary drug for cancer therapy, also caused myotube atrophy via blockage of ribosomal protein S6 kinase (p70S6K) signaling. Similarly, subjecting the DOX-treated myotubes to mechanical stretch counteracted the catabolic effect of DOX by restoring p70S6K activation (Guigni et al., 2019).

Another case of pathological muscle atrophy is caused by glucocorticoid therapy. Dexamethasone (Dex), a synthetic glucocorticoid, was shown to cause myotube atrophy *in vitro*. This was confirmed by the reduced total protein content and protein synthesis rate, decreased myotube diameter, as well as increased expressions of atrophy gene MuRF-1 and muscle atrophy factor box (MAFBx) (Chromiak and Vandenburgh, 1992; Aguilar-Agon et al., 2020). Excitingly, mechanical stretching either before or after treating myotubes with Dex mitigated all of these Dex-induced phenotypes (Chromiak and Vandenburgh, 1992; Aguilar-Agon et al., 2020). Moreover, the counteracting effect of mechanical stretch on Dex-induced myotube atrophy was abolished by prostaglandin synthesis inhibitor indomethacin, suggesting the involvement of prostaglandins (PGE2 and PGF2α) in counteracting Dex-induced myotube atrophy by stretch (Chromiak and Vandenburgh, 1994).

Therefore, mechanical stretch is generally accepted as a beneficial stimulus for skeletal muscle anabolism, not only by promoting myotube protein synthesis and hypertrophy, but also by counteracting the deleterious effects brought by glucocorticoid, cancer and its chemotherapy. In addition, skeletal muscle disuse- or overuse-induced atrophy was also reproduced by subjecting cultured myotubes to stretching cessation or overstretching. Overall, the protein turnover, hypertrophy and atrophy of myotubes under various stretching conditions were summarized in Table 1.

## Pathways Involved in Stretch-Induced Anabolism of Myotubes

### ERK

Extracellular regulated protein kinases (ERK) is a group of proteins regulating anabolism in many different tissues. The upstream events related to stretch-facilitated ERK phosphorylation and protein synthesis in myotubes included mechanical distortion of myotube membrane, activation of phospholipase A2 (PLA2) enzymes, and production of lysophospholipid (LPC) that could activate tyrosine kinases (Burkholder, 2009). Another upstream signaling of ERK in stretched myotubes was integrin-PP2A pathway, which could dephosphorylate ERK (Hanke et al., 2010). Even though ERK was dephosphorylated, the synthesis of fast myosin heavy chain (MHC-II) was upregulated in myotubes under such stretching condition (Hanke et al., 2010). Thus, ERK phosphorylation does not always play a decisive role in rendering anabolism of myotubes under mechanical strain. In support of this notion, Sasai et al. (2010) provided evidences that ERK exerted little effect on stretch-induced myotube hypertrophy, but instead was involved in attenuating myotube thickness under basal condition. Furthermore, hyperactivation of ERK negatively regulated anabolism of stretched myotube under some conditions. For example, conditioned media from lewis lung carcinoma (LLC) cells led to hyperactivation of ERK and concomitant inhibition of protein synthesis in stretched myotubes, which was restored by ERK inhibitor (Gao and Carson, 2016). Altogether, these diverse results reflected the bidirectional regulation of myotube anabolism by ERK under various stretching conditions.

### AKT

Protein kinase B (AKT) is another protein kinase for cell growth and protein synthesis. So far, almost all of the studies agreed with the positive role of AKT in promoting anabolism of myotubes under stretching stimuli. For instance, mechanical stretch stimulated myotube hypertrophy along with increased AKT phosphorylation (Sasai et al., 2010; Soltow et al., 2013; Aguilar-Agon et al., 2019), and blocking AKT pathway diminished myotube hypertrophy (Sasai et al., 2010). Conversely, cessation of stretch caused protein degradation and reduction in myotube size, concomitant with a decline in AKT phosphorylation (Soltow et al., 2013). However, Hanke et al. (2010) corroborated an opposing fact that AKT was dephosphorylated in stretched myotubes as a result of elevated phosphatase 2A (PP2A) activity, which was accompanied with strikingly raised expression of myofibrillar protein MHC-II. The reason for this controversial data is still unclear, but might result from different stretching patterns. In detail, the studies reporting positive role of AKT in rendering myotube anabolism applied cyclic or intermittent stretch (Sasai et al., 2010; Soltow et al., 2013; Aguilar-Agon et al., 2019), while negative role of AKT was shown during static stretch (Hanke et al., 2010).

### mTOR-p70S6K Pathway

Mammalian target of rapamycin (mTOR) is well-known for its control of cell growth and survival. By phosphorylating its substrate p70S6K, the mTOR-p70S6K pathway is associated with initiation of protein translation. Studies have shown that activation of mTOR-p70S6K pathway was positively correlated with stretch-induced anabolism of myotubes, such as increased protein synthesis and myotube diameter (Sasai et al., 2010; Nakai et al., 2015; Gao and Carson, 2016).

With regard to the mechanisms by which mechanical tension activate p70S6K pathway in myotubes, Lin and Liu (2019) reported that radial tension induced phosphatidic acid (PA)-enriched macropinosome formation in myotube membranes, which provided a platform for mTOR recruitment and activation. Consistently, another study reported that multiaxial stretch was able to induce p70S6K phosphorylation while uniaxial stretch was not (Hornberger et al., 2005). Taken together, we assumed the unique membrane deformation and cytoskeleton rearrangement by multiaxial or radial tension could promote macropinosome formation and subsequent activation of mTOR-p70S6K pathway in myotubes. In addition to the direct effect of stretching stimuli on p70S6K phosphorylation in myotubes, p70S6K could also be phosphorylated in stretched myotubes partially via an autocrine manner, because conditioned medium from stretched myotubes induced p70S6K phosphorylation in unstretched cells (Baar et al., 2000).

One common upstream node of ERK, AKT and mTOR pathways in stretch-stimulated myotubes is LRRC8A (leucine rich repeat containing 8 VRAC subunit A) that functionally comprises the volume-regulated anion channel (VRAC) (Kumar et al., 2020). Although LRRC8A expression was not altered in myotubes subjected to 5% static stretch, knock-down of the membrane bound LRRC8A led to impaired phosphorylation of ERK, AKT and mTOR pathways, possibly via its interaction with integrin (Kumar et al., 2020). Thus, LRRC8A channel complex could sense the mechanical stimuli and convert it into activation of these anabolic signaling pathways in myotubes.

### RhoA-AR Signaling

RhoA (ras homolog family member A) has been established as a crucial signaling effector mediating various cellular behaviors. Overexpression of active RhoA in myotubes led to increased protein synthesis and myotube growth via transactivation of androgen receptor (AR) (Lee et al., 2003). Moreover, mechanical stretch with low magnitude (1 and 2%) was able to induce both RhoA and AR protein expressions in myotubes, which were further potentiated by synchronized androgen stimulation (Lee et al., 2003; McClung et al., 2003). Thus, RhoA-AR signaling was a potential regulator of stretch-induced anabolism of myotubes, especially in the presence of androgen administration.

### Ang II Signaling

Angiotensin II (Ang II) is synthesized through enzymatic cleavage of angiotensinogen to form angiotensin I (Ang I), which is subsequently converted into Ang II by angiotensin-converting enzyme (ACE). Johnston et al. (2011) reported that mechanical tension led to significant elevations of both Ang II and its receptor AT2 (angiotensin II receptor type 2) in myotubes. The positive role of Ang II signaling in promoting protein synthesis has been well-demonstrated in other myogenic cell types such as vascular smooth muscle cells and cardiomyocytes (Lijnen and Petrov, 1999; Touyz et al., 1999). However, exogenous Ang II treatment of skeletal myotubes led to inhibited protein synthesis and elevated protein degradation (Sanders et al., 2005; Russell et al., 2006). Therefore, the activated Ang II signaling during mechanical stretching might negatively regulate anabolism of myotubes, even though this hypothesis was not tested by the authors (Johnston et al., 2011).

**Figure 2:**
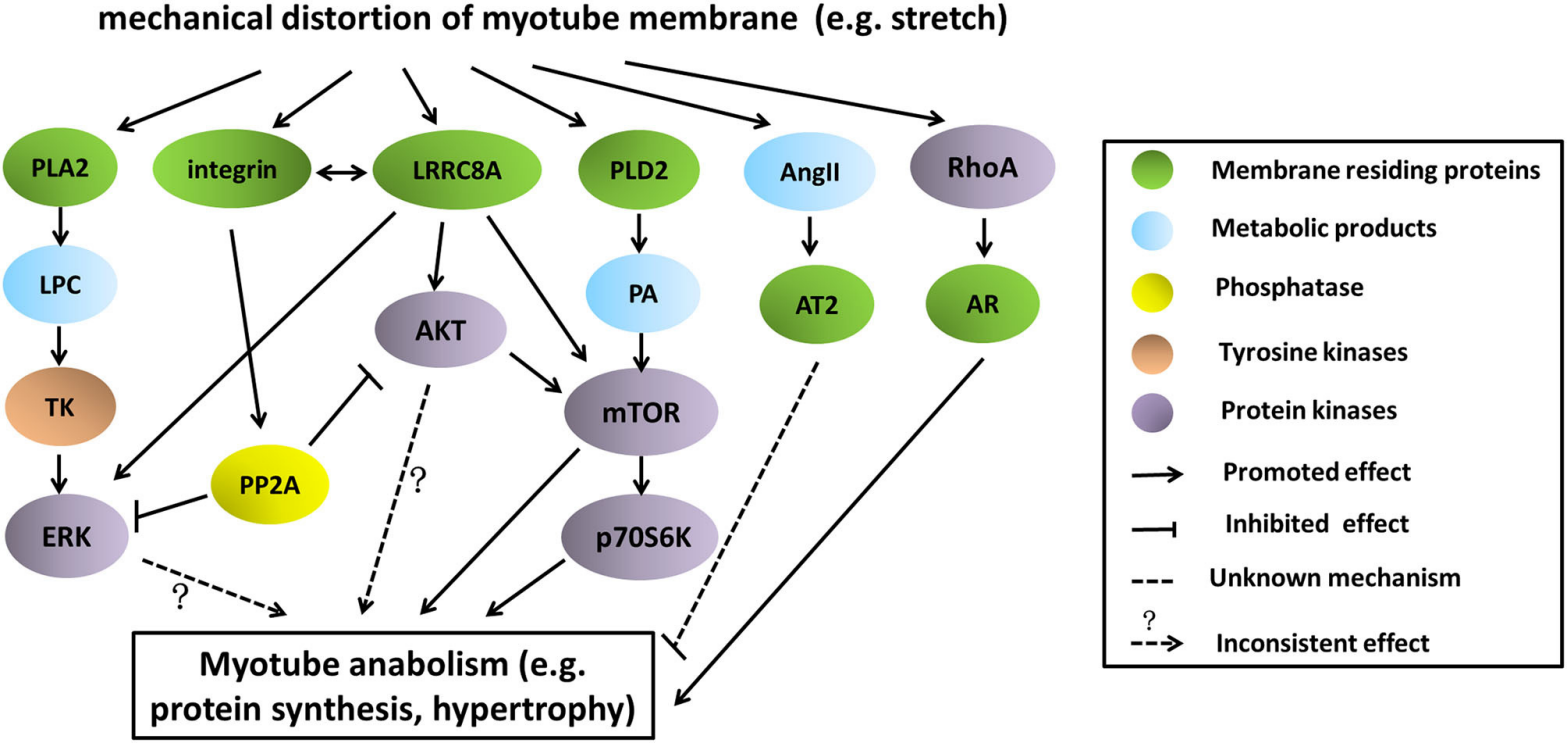
Pathways involving in stretch-induced myotube anabolism as well as their potential cross-talk. There were several pathways being proposed so far that mediate protein synthesis and myotube growth in stretching environment. Basically, stretching stimulated these pathways by distortion of myotube membrane, leading to activation of some membrane-residing proteins. Signaling molecules such as tyrosine kinases or phosphatase were involved in downward transmission, causing phosphorylation, or dephosphorylation of effector proteins (ERK, AKT, mTOR, p70S6K), and finally regulated the anabolism of myotubes.

Collectively, the depiction of these stretch-activated pathways involving in myotube anabolism as well as their potential cross talk was shown in Figure 2. It should be noted that the final consequences of protein turnover (either synthesis or degradation) in mechanically stretched myotubes largely depend on the mutual effects of anabolic and catabolic signaling, rather than activations of only some of them. In support of this, Atherton et al. (2009) demonstrated that stretch exerted catabolic functions in myotubes, despite activations of several anabolic pathways such as ERK, AKT, p70S6K.

## Glucose Transport

Exercises have been shown to be important for glycogen uptake in skeletal muscle (see review Evans et al., 2019). In consistent with the exercise-evoked glucose transport of muscle, some *in vitro* studies also manifested that cyclic stretch stimulated glucose uptake by cultured myotubes (Hatfaludy et al., 1989; Mitsumoto et al., 1992). The dynamics of glucose uptake were different among diverse myotubes (rat L6 myotubes vs. avian primary breast muscle myotubes) and distinct stretching regimes (magnitude, frequency, duration). However, both of the studies agreed that protein synthesis was necessary for stretch-induced glucose uptake in myotubes, since the protein synthesis inhibitor cycloheximide blunted this stimulatory effect of stretch (Hatfaludy et al., 1989; Mitsumoto et al., 1992). Furthermore, Chambers et al. (2009) investigated the potential pathways involved in transporting glucose into stretched myotubes. Their study focused on several pathways that have been implicated in glucose uptake of skeletal muscles: PI3K/AKT, AMP-activated kinase (AMPK), and p38 MAP kinase (p38MAPK). By using specific antagonists of these kinases, they demonstrated that p38MAPK was involved in stretch-induced glucose uptake of myotubes, while PI3K/AKT and AMPK were not (Chambers et al., 2009).

Insulin is also a well-known stimulator of glucose uptake. Whether mechanical tension and insulin promoted glucose transport into myotube via same or independent mechanisms still had not reached a conclusion. For example, Mitsumoto et al. (1992) insisted that insulin and stretch propelled glucose uptake through same pathways, since stretched myotubes and control myotubes had similar rate of glucose uptake when they were both treated with insulin. Conversely, research by Iwata et al. (2009) displayed an additive effect of insulin on stretch-incurred glucose uptake in myotubes, confirming that these two stimuli might activate independent pathways leading to glucose uptake.

It is noteworthy that Ca^2+^ played a critical role in mechanical signal transduction, and elevated intracellular Ca^2+^ level was accompanied with glucose transport in myotubes (Iwata M. et al., 2007; Iwata et al., 2009). Basically, there are two ways for intracellular Ca^2+^ mobilization: (1) Ca^2+^ influx from extracellular pools and (2) Ca^2+^ release from intracellular stores. Iwata et al. demonstrated that dantrolene, an inhibitor of Ca^2+^ efflux from the sarcoplasmic reticulum, could abrogate stretch-induced glucose uptake in myotubes, while extracellular Ca^2+^ depletion by egtazic acid (EGTA) could not. Therefore, it was asserted that mechanical tension promoted intracellular Ca^2+^ mobilization via increased Ca^2+^ releasing from sarcoplasmic reticulum in myotubes, which activated downstream pathways such as Ca^2+^/calmodulin-dependent kinase (CaMK) that participated in glucose uptake (Iwata M. et al., 2007; Iwata et al., 2009).

The direct mediators in regulating glucose influx into muscle cells are glucose transporter (GLUT) isoform 1 and 4. During the process of myogenesis, the expression of GLUT1 decreased but GLUT4 increased, which account for the fact that mechanical stretch had no effect on glucose uptake by myoblasts but significantly promoted glucose uptake by myotubes (Mitsumoto et al., 1992; Iwata M. et al., 2007). Interestingly, glucose uptake into stretched myotubes was independent of GLUT4 protein level but rather dependent upon its translocation to the plasma membrane (PM) (Mitsumoto et al., 1992; Nedachi et al., 2009; Saito et al., 2016). Two mechanisms had been proposed to orchestrate GLUT4 mobilization in myotubes. One was that mechanical stretch led to production and secretion of C-X-C motif chemokine ligand (CXCL) 1 and 5, which further affected GLUT4 PM translocation by binding to C-X-C motif chemokine receptor 2 (CXCR2) (Nedachi et al., 2009). The other one involved Adaptor protein, Phosphotyrosine interaction, Pleckstrin homology domain, and Leucine zipper containing 1 (APPL1). In fact, mechanical stress promoted PM translocation of APPL1, which was necessary for GLUT4 disassociation with protein phosphatase 2A (PP2A) and subsequent interaction with myosin II ATPase on PM (Saito et al., 2016). Altogether, using C2C12 myotubes, these studies encompassed possible pathways involved in stretch-induced GLUT4 translocation and glucose uptake. Whether these molecular basis are still applicable to myotubes from different species remains to be explored in future.

## Myotube Maturation

The *in vitro* formation of myotubes is usually induced by culturing myoblasts in differentiation medium. More than affecting the initial differentiation of myoblasts, mechanical stretching also influences the maturation of myotubes. The terminally differentiated and mature myotubes are characterized by the appearance of sarcomeric striated structures such as A bands and Z lines that are formed by contractile proteins myosin heavy chain (MHC) and α-actin, respectively. For example, Liao et al. (2008) and Bansai et al. (2019) observed more concentrated α-actin formed in mechanically stretched myotubes, which had a higher ratio of mature to immature myotubes than non-stretched myotubes.

Cyclic stretching could also upregulate MHC protein expression in myotubes (Liao et al., 2008; Candiani et al., 2010). Moreover, study by Huang et al. (2012) showed that stretch increased myotube MHC mRNA expression in stretching frequency- and duration-dependent manners. Through applying a series of stretches with different frequency (0, 0.25, 0.5, and 1Hz) and duration (5, 7, and 10 day), the authors reported that the highest expression of MHC occurred on frequency 0.25 Hz at day 7. With respect to the mechanisms of stretch-induced MHC expression in myotubes, Rauch and Loughna (2005) proposed that short-term (from minutes to hours) static stretch caused myocyte enhancer factor 2A (MEF2A) dephosphorylation and nuclear translocation, which transactivated neonatal MHC (MHCneo) isoform. However, their later study applying long-term static stretch (3 days) demonstrated that elevated levels of MHC fast and slow isoforms (MHCf and MHCs) were not attributed to MEF2A phosphorylation (Rauch and Loughna, 2006).

Strikingly, study by De Deyne (2000) illustrated that uniaxial stretch could neither inhibited nor facilitated the appearances of Z lines or A bands in myotubes. In contrast, they found a positive correlation between these sarcomeric striated structures with electrical stimulation (De Deyne, 2000). In addition, another similar study applying mechanical, electrical, or combination of both stimuli on myotubes demonstrated that synchronized electromechanical stimuli led to higher levels of contractile proteins and higher percentage of mature myotubes than either stimuli alone (Liao et al., 2008). The reason for these discrepancies in stretch-induced myotube maturation was unclear, but might possibly result from different experimental conditions, such as myotube differentiation status, culturing conditions, and stretching parameters.

## Myotube Alignment

*In vivo*, the muscle fibers lie in line with the direction of muscular movement to be fully functional in producing forces. The alignment of *in vitro* cultured myotubes was also affected by mechanical stretching. Using apparatus stretching myotubes radially, Vandenburgh et al. found that continuous stretching for 3 days resulted in increased percentage of myotubes orienting in the direction of stretch. Moreover, the alignment of myotubes relied on the rate of stretch (from 0.35 mm/h to 0.07 mm/h), with the rate of 0.21 mm/h exerting the most obvious reorienting effect (Vandenburgh, 1982). While continuous radial stretch promoted the parallel alignment of myotubes along the stretching axis, intermittent radial stretching stimuli reoriented myotubes perpendicularly to the stretching direction (Vandenburgh, 1988). Altogether, these studies proved that radial stretch affected myotube alignment in a stretching rate dependent manner.

On the other hand, uniaxial stretch at the rate of 0.35 mm/h also aligned the myotubes parallel to each other along the axis of stretch, which was accompanied by elongation of myotubes (Vandenburgh et al., 1989). Similarly, another study by Collinsworth et al. displayed equally evident parallel reorientation and longitudinal growth of myotubes under uniaxial stretching at rate of either 0.048 mm/h or 0.036 mm/h. Therefore, it seemed that stretching rate did not affect the alignment efficiency in uniaxially stretched myotubes (Collinsworth et al., 2000).

In conclusion, even though both radial and uniaxial stretch could reorient myotubes, stretching rate might play different roles in their alignment. However, the mechanisms of stretch-induced reorientation of myotubes were not discussed in these studies, and await further exploration.

## Cytoskeletal Adaption

Cytoskeletal proteins such as actin, vinculin, and talin mediate cell spreading and elasticity. When upregulated by mechanical stimuli, these proteins may stabilize the cytoskeleton, thus enabling cells to bear mechanical stress (see review Hirata et al., 2014). The promoter activity and mRNA level of α-actin were repressed by 6–24 h static stretch in myotubes. This was in sharp contrast to accumulation of α-actin protein in stretched myotubes, which could be due to either increased protein synthesis or decreased protein degradation. Thus, we assumed that both translational and post-translational regulations could be involved in stretch-elevated α-actin protein in myotubes (Carson and Booth, 1998). Other cytoskeletal elements, such as talin and vinculin, were also reported to be synthesized in stretched myotubes. Upon cyclic mechanical stimuli, both mRNA and protein levels of talin and vinculin were elevated in myotubes (Frenette and Tidball, 1998; Tidball et al., 1999). By measuring apparent elastic modulus (E_app_) of the myotubes, Zhang et al. (2004) found positive correlation between E_app_ and expressions of vinculin and talin in stretched myotubes. In addition, turnover rate of talin protein has been reported to be accelerated by mechanical stretch, as shown by increased quantities of both intact talin and its 190-kDa proteolytic fragment (Frenette and Tidball, 1998). Thus, studies above manifested that myotubes responded to mechanical stretching by stimulating expressions of these cytoskeletal proteins, aiming to maintain the cytoskeleton, and withstand mechanical distortion.

Regarding the mechanisms of stretch-induced talin and vinculin expressions in myotubes, Tidball et al. (1998, 1999) proposed the involvement of nitric oxide (NO), which was produced and released by myotubes during cyclic stretching. NO modulated vinculin and talin at the mRNA level by influencing the rate of their transcription via cGMP-dependent protein kinase G (PKG) pathway. The role of NO-PKG axis in promoting talin and vinculin expressions was further confirmed by using NO donor and scavenger, as well as PKG inhibitor (Tidball et al., 1999). Calpain is one of the major proteolytic enzymes that could cleave intact vinculin and talin proteins into proteolytic segments. Intriguingly, Soltow et al. (2013) verified that both stretch-induced intact talin protein level and cessation of stretch-induced cleaved talin protein level were dependent upon NO production, suggesting a dual role of NO that could either inhibit calpain proteolysis in stretched myotubes or activate calpain proteolysis during cessation of stretch (Zhang et al., 2004).

The dystrophin-associated glycoprotein complex (DGC) has been shown to regulate NO via many different signaling. Notably, the dynamics of stretch-released NO was significantly altered in myotubes from dystrophin deficient mdx mice (Wozniak and Anderson, 2009). Thus, when myotubes are subjected to mechanical stress, functional DGC might be necessary in modulating NO production, and subsequently in promoting the cytoskeletal adaption of myotubes through synthesis of cytoskeleton proteins.

## Myotube Damage

One of the early changes of myotubes subjected to mechanical stretch is membrane distortion accompanied with increased permeability. Burkholder (2003) compared the effect of cyclic stretch with different amplitudes and frequencies on myotube membrane permeability by detecting fluorescein labeled dextran (FDx) dye uptake. Their study illustrated that at amplitude within the physiological range, dye uptake occurred in a punctate pattern independently of stretching frequency. When the amplitude was larger than 15%, frequency became the dominating factor and the pattern of dye uptake became diffuse, reflecting transient disruption of cell membrane (Burkholder, 2003). In contrast, little damage was found in myotubes subjected to cyclic stretch with 10% amplitude at frequency of 10 cycles/min, which was confirmed by few vacuole formation in cytoplasm of stretched myotubes (Adachi et al., 2003). The stretching amplitude and frequency-dependent myotube damage was also shown in other studies that quantified the extent of myotube damage through measurement of creatine kinase (CK) activity and lactate dehydrogenase (LDH) release in culture media (Vandenburgh et al., 1989, 1990; Peterson and Pizza, 2009).

Does the stretch-induced myotube damage have some physiological significance? We assumed the answer is yes if myotubes are normal and healthy. In support of this notion, many studies had verified that short-term stretch-provoked myotube damage was transient and accompanied with myotube anabolism and growth (Atfaludy et al., 1988; Vandenburgh et al., 1989, 1990; Clarke and Feeback, 1996). For example, the increased permeability of myotube membrane provided convenient influx of exogenous factors and efflux of endogenous factors, which might contribute to myotube protein synthesis and hypertrophy (Vandenburgh et al., 1989). As had been mentioned before in this review, FGF was one of these endogenous factors released from stretch-wounded myotubes and prompted myotube protein synthesis in an autocrine manner (Clarke and Feeback, 1996). In addition, stretch-induced prostaglandins (such as PGE2 and PGF2) efflux as a result of membrane disruption also mediated the protein turnover in myotubes (Vandenburgh et al., 1990).

## Relationship Between Myotube Structural Proteins and Stretch-Induced Damage

As discussed before, cytoskeleton of normal myotubes could adapt to mechanical stretch. Therefore, stretch-induced damage in these myotubes are transient and even beneficial for their metabolism and growth. However, severe disorders occurred when some structure-related genes are depleted or mutated, which disabled myotubes to reconstruct their cytoskeleton in order to withstand the mechanical disruption. Some of these structural genes in enabling myotubes to resist mechanical stretching are exemplified in the below.

### Caveolin-3

Caveolae are cup-shaped invaginations with 60–80 nm in diameter that are present at the plasma membrane of many cells, especially in muscle cells, adipocytes, and endothelial cells. Caveolin-3, the major constituent of the caveolar structure, is specifically expressed at the membrane of skeletal muscle cells and associated with endocytosis and cell signaling in response to mechanical stimuli (Bellott et al., 2005; Dewulf et al., 2019).

Caveolae function as membrane-mediated sensors and regulators of the plasma membrane tension. Sinha et al. (2011) demonstrated that myotubes under stretching stimuli had flattened caveolae, which is beneficial for buffering membrane tension surges during mechanical stress. When mechanical stress was terminated, caveolae returned to their normal state. Notably, the absence of a functional caveolae in myotubes resulting from caveolin-3 depletion or mutation displayed enhanced membrane fragility and rupture under mechanical stress, supporting the role of caveolin-3 as an accommodator of external mechanical stimulation (Dewulf et al., 2019).

### Sarcoglycan

The actin cytoskeleton and the extracellular matrix are linked via dystrophin and dystrophin-associated proteins (DAPs). Being components of DAPs, sarcoglycan proteins are important in regulation of mechanosensitive signaling in myotubes. For instance, Moorwood et al. (2014) confirmed γ-sarcoglycan as a regulator of stretch-activated p70S6K signaling in myotubes. Mechanical stretch only caused a transient elevation of p70S6K phosphorylation in normal myotubes, but p-p70S6K level sustained in γ-sarcoglycan^−/−^ myotubes subjected to mechanical stimuli (Moorwood et al., 2014).

Dystrophin-DAP complex is vital in maintaining mechanical stability of the sarcolemma, the dysfunction of which led to inability of myotubes to withstand mechanical stress and resultant myotube damage. Shigekawa et al. demonstrated that disrupted Ca^2+^ homeostasis resulting from hyperactivated Ca^2+^ channels was the fundamental mechanism of the susceptibility of sarcoglycan^−/−^ myotubes to stretch-induced damage (Nakamura et al., 2001; Sampaolesi et al., 2001; Iwata et al., 2003). These Ca^2+^ permeable channels could be activated by mechanical stimulation in a controllable manner when myotubes possess functional dystrophin-DAP complex. However, sarcoglycan deficiency in myotubes caused abnormal hyperactivation of these channels, which was further aggravated upon mechanical stretching. Therefore, Ca^2+^ influx was dramatically strengthened when sarcoglycan deficient myotubes were subjected to mechanical stress, incurring to calpain-mediated cytoskeleton degradation, membrane rupture, and cell damage (Sampaolesi et al., 2001).

It is noteworthy that these Ca^2+^ permeable channels might be activated simultaneously or sequentially when sarcoglycan deficient myotubes were stretched. According to these studies, the stretch-activated cation channels (SACs) that were hyperactivated in sarcoglycan deficient myotubes were possibly the primary cause of Ca^2+^ influx under stretch (Nakamura et al., 2001). This initial Ca^2+^ accumulation in myotubes provoked the membrane depolarization, secondarily activating voltage-gated Ca^2+^ channels such as L-type Ca^2+^ channel (Sampaolesi et al., 2001). Alternatively, the third way of Ca^2+^ influx was via growth factor–regulated channel (GRC) translocation to the sarcolemma of myotubes, which could be facilitated by both stretch and sarcoglycan depletion (Iwata et al., 2003). Consistent with the notion that Ca^2+^ overloading made sarcoglycan deficient myotubes less endurable to mechanical stress, specific inhibitors of these Ca^2+^ channels (SACs, L-type Ca^2+^ channel, GRC) such as GdCl_3_, nifedipine, ruthenium red, as well as extracellular Ca^2+^ chelation BAPTA, all effectively attenuated stretch-induced damage in sarcoglycan deficient myotubes (Nakamura et al., 2001; Sampaolesi et al., 2001; Iwata et al., 2003).

In contrast to the increased Ca^2+^ influx into sarcoglycan deficient myotubes, Iwata Y. et al. (2007) proposed another mechanism through which decreased Ca^2+^ efflux out of sarcoglycan deficient myotubes also propelled Ca^2+^ overloading and myotube damage during mechanical stretching. In detail, Adenosine Triphosphate (ATP) was found to be released continuously from sarcoglycan deficient myotubes in a manner further stimulated by stretching. Autocrine ATP release evoked activation of Na^+^/H^+^ exchanger (NHE), leading to increased intracellular Na^+^ level. Subsequently, high intracellular Na^+^ caused reduced Ca^2+^ extrusion via Na^+^/ Ca^2+^ exchanger (NCX), finally conducing to Ca^2+^ overloading (Iwata Y. et al., 2007). The ATP-NHE-NCX axis in mediating stretch-induced sarcoglycan deficient myotube damage was well-testified by blocking ATP, NHE, and NCX using ATP hydrolyzing enzyme apyrase, cariporide, and KB-R7943.

### Plectin

Plectin, a giant cytolinker protein, plays a crucial role in stabilizing and orchestrating intermediate filament (IF) networks in cells. Plectin-deficient myotubes were characterized by aggregations of desmin protein and disarrangement of the myofibrillar apparatus. Particularly, when plectin-deficient myotubes were subjected to 30% stretch at 0.25 Hz for 1 h, there were significantly more cells detached than control cells, indicating lower resilience toward mechanical stretch (Winter et al., 2014). The authors assume that these abnormalities in plectin-deficient myotubes were at least partially related with increased expressions of heat shock proteins (HSPs), since chemical chaperon 4-phenylbutyrate (4-PBA) led to increased mechanical resilience of plectin-deficient myotubes.

**Figure 3:**
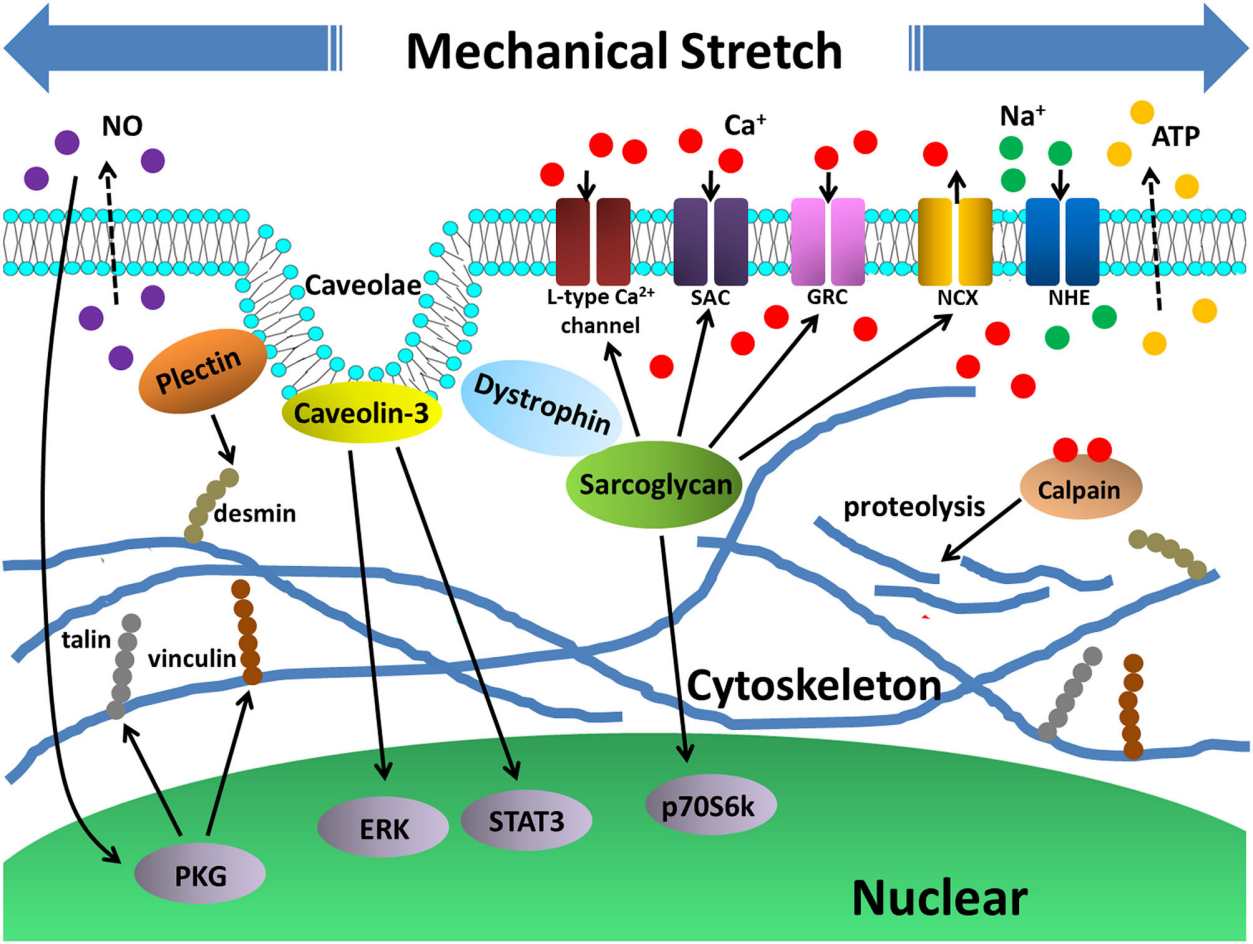
Stretch-induced myotube cytoskeletal reconstruction and cellular damage. Cytoskeletal proteins such as sarcoglycan, plectin, as well as membrane caveolae protein caveolin-3 are all critical proteins that participated in myotube cytoskeleton adaptation to mechanical stretch by activating signalings such as p70S6K, STAT3, ERK. In addition, release of NO also played a role in activating PKG pathway. The activation of these pathways contributed to expressions of structure proteins desmin, talin and vinculin, which are beneficial to reconstructions of myotube cytoskeleton. On the other hand, deficiency of the sarcoglycan led to abnormal activities of Ca^2+^ and Na^+^ channels, which caused intracellular Ca^2+^ overloading and activation of Ca^2+^ dependent hydrolase, calpain, that promoted cytoskeletal protein proteolysis and cellular damage.

Taken together, normally functioned myotubes are able to resist mechanical stretch through cytoskeletal adaptive reconstruction. Conversely, stretch-induced myotube damage occurs when some critical structure-related proteins are depleted or mutated, leading to disrupted cell homeostasis (Figure 3).

## Secretion of Cytokines

**Figure 4:**
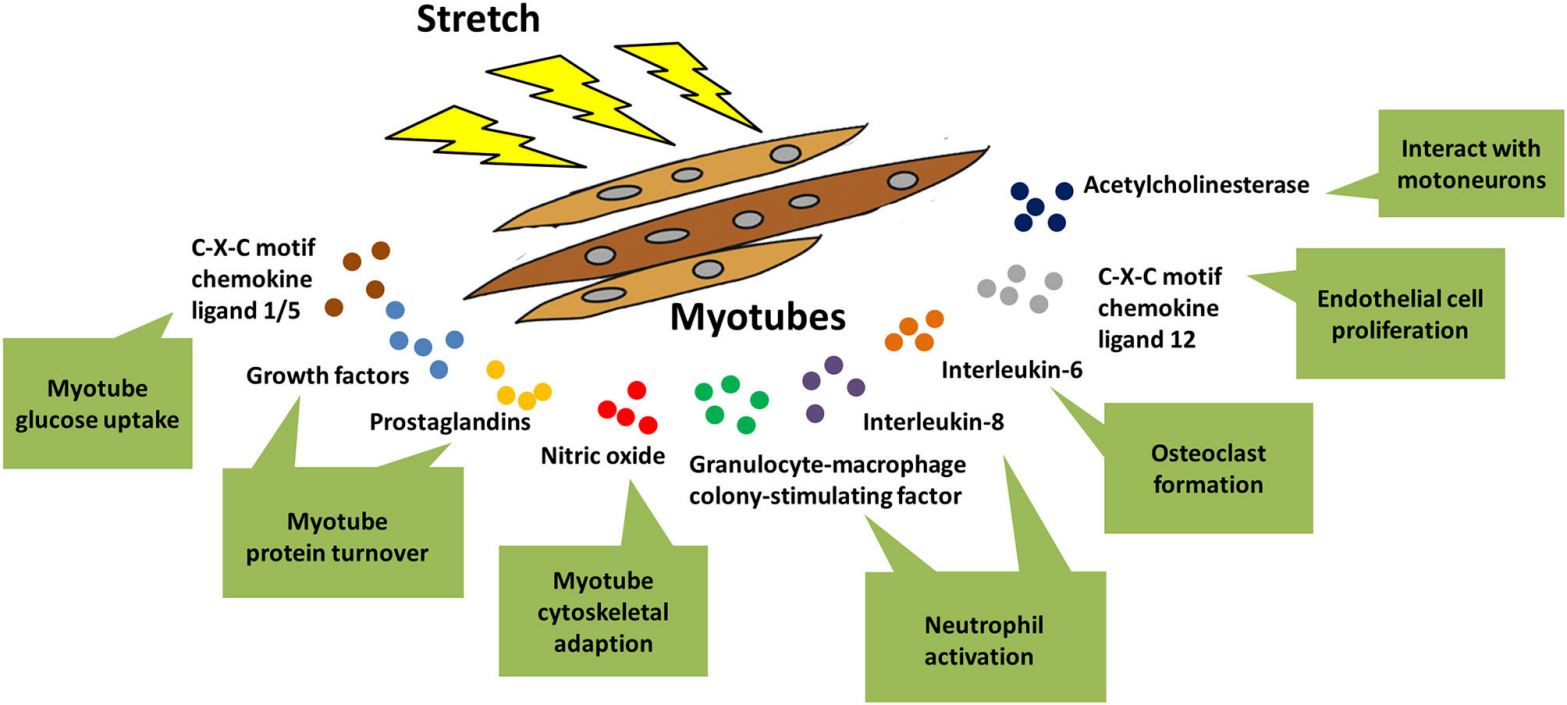
Stretch-regulated secretion of myotubes. When myotubes were stimulated by mechanical stretch, various kinds of cytokines were released out of myotubes and exerted distinct functions. Some of these cytokines acted in an autocrine fashion, regulating glucose uptake, protein synthesis, and cytoskeletal adaption of myotubes in response to mechanical stretch. In addition, there were other cytokines that were involved in cross-talk of stretched myotubes with other types of cells, such as neutrophil, motoneurons, osteoclasts, and endothelial cells.

Skeletal muscle is a secretory organ that could excrete cytokines through autocrine, paracrine, and endocrine modes. Accumulating evidences pointed out that myotubes are the main sources of cytokine production in skeletal muscle. Some of these cytokines (IGF-1, FGF, PGE2, PGF2α, CXCL1, CXCL5, NO) acted in an autocrine fashion, regulating the protein turnover, glucose uptake, and cytoskeletal adaption of myotubes in response to mechanical stretch, as had been discussed in detail in the previous parts of this review (Vandenburgh et al., 1990; Perrone et al., 1995; Clarke and Feeback, 1996; Tidball et al., 1998, 1999). Other cytokines functioned in paracrine or endocrine fashions, and were involved in the interactions of stretched myotubes with distinct cell types in the below (Figure 4).

### Motoneurons

Acetylcholinesterase (AChE) is an essential component of cholinergic synapses in both central and peripheral nervous systems, since it is responsible for the rapid hydrolysis of acetylcholine (ACh) released from presynaptic nerve terminals (Massoulie et al., 1993). Hubatsch and Jasmin (1997) reported that stretched myotubes could secret the G4 form of AChE, possibly through transcriptional activation of AChE mRNA by Egr-1 (early growth response 1). Thus, it is plausible that skeletal muscle fibers interact with motoneurons to regulate AChE expression in the neuromuscular junctions that undergo intense mechanical distortions during exercise.

### Neutrophils

Neutrophils accumulate in skeletal muscles being exposed to mechanical loading, in order to erase the damaged muscle fibers at the sites of inflammation. *In vitro* activation of neutrophils, including neutrophil chemotaxis and priming for ${\text{O}}_{2}^{-}$ production, was significantly promoted by the conditioned medium from stretched myotubes (Tsivitse et al., 2005; Peterson and Pizza, 2009). Different cytokines that were secreted from myotubes upon distinct stretching parameters mediated neutrophil activation. For example, application of intensive strain (0.25 Hz, 15% magnitude) for a shorter period of time (30 min) resulted in secretion of monocyte chemotactic protein 1 (MCP-1), granulocyte-macrophage colony-stimulating factor (GM-CSF), Interleukin-6 (IL-6), Interleukin-8 (IL-8), and granulocyte colony-stimulating factor (G-CSF) from myotubes, with IL-8 and GM-CSF contributing to neutrophil activation (Peterson and Pizza, 2009). However, utilizing a less drastic strain (0.25 Hz, 5 and 10% magnitude) but for a longer duration (2 h) inhibited secretion of IL-8, tumor necrosis factor-α (TNF-α) and transforming growth factor-β (TGF-β) from myotubes, thus excluding their involvements in prompting neutrophil activation (Tsivitse et al., 2005).

In addition, stretch caused a more complex interaction between co-cultured myotubes and neutrophils. On one hand, mechanical loading of myotubes increased the release of myeloperoxidase (MPO) from co-cultured neutrophils, implying stretched myotubes mediated neutrophil activity via paracrine manners. On the other hand, co-cultured neutrophils greatly enhanced stretch-induced myotube damage (Nguyen et al., 2005). Collectively, myotubes seemed to promote their own lysis during mechanical tension by cross-talking with neutrophils.

### Osteoclast

Muscle-derived factors have been shown to regulate bone formation and resorption, implying their roles in maintaining the musculoskeletal homeostasis. Juffer et al. (2014) found that conditioned medium from non-stretched myotubes had a inhibitory effect on osteoclast formation, while the conditioned medium from stretched myotubes promoted osteoclast formation. The authors further identified elevated IL-6 secretion from myotubes under mechanical tension, which partially accounted for the propelling effect on osteoclast formation (Juffer et al., 2014).

### Endothelial Cell

Capillaries are essential for the supply of oxygen and nutrients to skeletal muscle during exercise. Some muscle-derived factors were reported to affect the angiogenesis of capillaries. For example, the chemokine stromal cell derived factor-1 (SDF-1α, also known as CXCL12) was associated with angiogenesis under physiological and pathological states (Xu et al., 2013). Notably, both mRNA and protein levels of CXCL12 were enhanced in myotubes subjected to 24 h stretching, but not in myoblasts under same stimulation. Moreover, mechanical stretch-released CXCL12 from myotubes prompted vascular endothelial cell proliferation via binding to CXC receptor 4 (CXCR4) on endothelial cells, as both CXCL12 neutralizing antibody and CXCR4 antagonist abolished endothelial cell proliferation (Yamada et al., 2019). Despite of the *in vitro* effect of CXCL12-CXCR4 signaling axis on endothelial cell proliferation, muscle-specific CXCL12 knockout mice displayed normal angiogenesis when they exercised, suggesting CXCL12 might be a redundant factor in preserving exercise training-mediated angiogenesis in skeletal muscles (Yamada et al., 2019).

## Stretch-Activated Ion Channels in Myotubes

Mechanical distortion of myotube membrane inevitably affect the activities of ion channels present on membrane. Some cation channels, such as Na^+^ pump and Ca^2+^ channel, were easily activated in myotubes subjected to various stretching conditions, and participated in mechanoresponses of myotubes. The involvements of these ion channels in stretch-induced amino acid uptake, glucose transport, protein synthesis, secretion, and damage of myotubes have already been discussed in previous parts of this review, and will be briefly summarized in Table 2.

**Table 2:** Involvement of stretch-activated ion channels on biomechanical manifestations of myotubes.

**Effects of stretch on myotubes**	**involved ion channels**	**Activity of ion channels**	**Functional analysis**	**References**
Increased amino acid uptake	Na pump	Increased Na pump activity	Inhibited by ouabain (depolarize the myotube's membrane potential)	Vandenburgh, 1983
Increased amino acid uptake	Na pump	Not reported	Inhibited by ouabain and tetrodotoxin (inhibit sodium influx of an electrogenic nature) inhibit stretch-induced amino acid uptake, but not by amiloride (inhibit sodium influx of non-electrogenic nature)	Drummond et al., 2010
Increased glucose transport	Na pump is not involved	Not reported	Not inhibited by tetrodotoxin	Johnston et al., 2011
Increased glucose transport	Ca channel is not involved	Not reported	Not inhibited by reducing extracellular calcium level	Evans et al., 2019
Increased protein synthesis	Na pump is not involved	Not reported	Not inhibited by tetrodotoxin	Clarke and Feeback, 1996
Increased prostaglandins secretion	Na pump is not involved, but Ca channel might be	Not reported	Not inhibited by tetrodotoxin, but inhibited by reducing extracellular calcium level	De Deyne, 2000
Increased prostaglandins secretion	Na pump is not involved	Not reported	Not inhibited by tetrodotoxin	Vandenburgh, 1982
Increased acetylcholinesterase secretion	Na pump is not involved, but L-type Ca channel is involved	Not reported	Not inhibited by tetrodotoxin, but inhibited by nifedipine (L-type Ca channel inhibitor)	Tidball et al., 1998
Increased damage of sarcoglycan deficient myotubes	L-type Ca channel is involved	Not reported	Inhibited by nifedipine and tranilast (inhibit growth factor-induced Ca influx)	Hubatsch and Jasmin, 1997
Increased damage of sarcoglycan deficient myotubes	Stretch-activated cation channel (SAC) is involved	Increased SAC activity	Inhibited by Gadolinium chloride (GdCl3, stretch-activated channel inhibitor)	Tsivitse et al., 2005
Increased damage of sarcoglycan deficient myotubes	Growth factor-regulated channel (GRC) is involved	Not reported	Inhibited by transfection with GRC antisense cDNA	Nguyen et al., 2005
Increased damage of sarcoglycan deficient myotubes	Na+/H+ exchanger (NHE) and Na^+^/Ca^2+^ exchanger (NCX) are involved	Increased NHE activity	Inhibited by EIPA and cariporide (NHE inhibitors), and KBR (NCX inhibitor)	Juffer et al., 2014
Activation of anabolical pathways (AKT, ERK, mTOR)	Volume-regulated anion channel (VRAC) is involved	Not reported	Inhibited by VRAC component LRRC8A ablation	Kumar et al., 2020

## Oxidative Stress

Myotubes experienced endogenous oxidative stress when they were subjected to mechanical stimuli. Upon exposure to 15% stretch for 10 min, the intracellular reactive oxygen species (ROS) levels raised moderately, contributing to glucose uptake by myotubes (Chambers et al., 2009). However, a more intensive stretching protocol, such as 18% stretch for 12 h, led to dramatic generation of ROS in myotubes, which caused increased oxidative damage of DNA, reduced myotube diameter and MHC intensity (Kim et al., 2018).

Ebihara et al. (2002) explored the sarcolemmal injury in cultured L6 myotubes induced by oxidative stress, mechanical stretch, or both of them. Their data suggested that oxidative stress greatly increased the vulnerability of myotubes to stretch-induced sarcolemmal injury. Considering that membrane permeability of myotubes was increased upon sarcolemmal damage, it should be interesting to investigate whether ROS generated during stretch will be released into extracellular matrix and acted as exogenous oxidative stress. Presumably, this positive feedback between mechanical and oxidative stress could have made myotubes more susceptible to stretching stimuli and resulted into synergetic mechanical-oxidative stress-induced myotube damage.

In prevention of unlimited ROS accumulation during stretching, myotubes possess antioxidative systems that could scavenge ROS and protect themselves from sarcolemmal damage. By exposing C2C12 myotubes to 30 min of 15% stretch, Pardo et al. (2011) proposed a mechanism by which an antioxidative signaling was simultaneously activated in myotubes to counteract ROS overproduction. Specifically, mechanical stretch promoted transient elevation of EGR1 (early growth response factor 1), which transactivated sirtuin1 (SIRT1) and subsequently induced manganese superoxide dismutase (MnSOD) that contributed to elimination of excessive ROS. Interestingly, an autoregulatory loop existed in this antioxidative system consisting of EGR1 and SIRT1 (Pardo and Boriek, 2012). These two proteins tended to physically interact when SIRT1 reached maximal level, and the EGR1-SIRT1 protein complex had a prohibitory role on the activation of the Sirt1 promoter. Through this autoregulatory loop, EGR1 and SIRT1 protein levels could return to their initial states when ROS was diminished to basal levels. Thus, this negative-feedback mechanism allowed myotubes to maintain oxidative homeostasis when they were constantly exposed to stretching stimuli (Pardo and Boriek, 2012).

## Discussion

So far, various stretching regimes were reported to provoke distinct mechanoresponses of myotubes, such as metabolism, growth, secretion, cytoskeleton reconstruction, damage, oxidative stress. However, most studies only focused on one or a few aspects, and neglected the interconnections among them. Therefore, one challenge in future study is systematically investigating all of these biomechanical alterations of myotubes under some stretching condition, as well as penetrating potential correlations among these changes. In order to do so, microarray analysis will be a powerful tool to give us a global view of how myotubes respond to different types of stretch, rather than the examination of separate molecules and pathways as had been done before.

Apart from stretching condition, the differentiation or maturation status of myotubes is also crucial in determining their mechanoresponses. Up to date, *in vitro* cultured myotubes are all derived from induced differentiation of myoblasts. This inevitably leads to mixed groups of myotubes with various levels of maturation, as well as undifferentiated myoblasts, which makes it impossible to attain myotubes at the same level of maturation. Thus, future development of single myotube *in vitro* culturing and stretching will provide us a better model to accurately study biomechanical adaptions of myotubes. Moreover, how could the maturation status of myotubes affect their mechanoresponses is currently unknown and awaits further elucidation through application of this model.

*In vivo*, myotubes are not the only cells subjected to mechanical tension during muscle stretching. Another substantial group of cells being simultaneously stretched is myoblasts or satellite cells, the progenitors of myotubes. Notably, myoblasts responded to stretching stimuli in many different aspects comparing to myotubes, as had been reviewed before (Wang et al., 2020). In detail, mechanical stretch had been reported to influence proliferation, differentiation, and pluripotency of myoblasts, while the metabolisms of myotubes such as amino acid and glucose uptake, protein turnover, hypertrophy, and secretion were affected by stretching stimulations. However, both cells could experience cytoskeletal reconstruction and cellular reorientation in response to proper mechanical stimuli. Moreover, they both suffer damage if stretching is too intense to resist or there were some deficient genes that disabled cytoskeletal adaptions to external stretching. Overall, we could assume that when skeletal muscles are passively elongated, myoblasts and myotubes jointly contribute to skeletal muscle mechanobiology. Thus, it will be interesting to study the cross talk between *in vitro* cultured myoblasts and myotubes in response to mechanical stretch, which could greatly simulate *in vivo* muscular stretching microenvironment.

## Author Contributions

DR had the idea of this review and prepared the original draft. JS and RL performed the literature search. XZ made the tables and figures of this review. XYu made some valuable suggestions and corrections to this review. XYa and QZ participated in the revision process. All authors contributed to the article and approved the submitted version.

## Conflict of Interest

The authors declare that the research was conducted in the absence of any commercial or financial relationships that could be construed as a potential conflict of interest.

## Publisher's Note

All claims expressed in this article are solely those of the authors and do not necessarily represent those of their affiliated organizations, or those of the publisher, the editors and the reviewers. Any product that may be evaluated in this article, or claim that may be made by its manufacturer, is not guaranteed or endorsed by the publisher.

## References

[B1] Aas V. Bakke S. S. Feng Y. Z. Kase E. T. Jensen J. Bajpeyi S.. (2013). Are cultured human myotubes far from home? Cell Tissue Res. 354, 671–682. 10.1007/s00441-013-1655-1 23749200

[B2] Adachi R. Yabusaki K. Obinata T. (2003). Uptake of albumin is coupled with stretch-induced hypertrophy of skeletal muscle cells in culture. Zool. Sci. 20, 557–565. 10.2108/zsj.20.557 12777827

[B3] Aguilar-Agon K. W. Capel A. J. Fleming J. W. Player D. J. Martin N. R. W.. (2020). Mechanical loading of tissue engineered skeletal muscle prevents dexamethasone induced myotube atrophy. J. Muscle Res. Cell Motil. 10.1007/s10974-020-09589-0. [Epub ahead of print]. 32955689 PMC8332579

[B4] Aguilar-Agon K. W. Capel A. J. Martin N. R. W. Player D. J. Lewis M. P. (2019). Mechanical loading stimulates hypertrophy in tissue-engineered skeletal muscle: Molecular and phenotypic responses. J. Cell Physiol. 234, 23547–23558. 10.1002/jcp.28923 31180593 PMC6771594

[B5] Atfaludy S. Shansky J. Vandenburgh H. H. (1988). Skeletal muscle cell growth and creatine kinase release during stretch relaxation activity in tissue culture. Can. J. Sports Sci. 13:15.

[B6] Atherton P. J. Szewczyk N. J. Selby A. Rankin D. Hillier K. Smith K.. (2009). Cyclic stretch reduces myofibrillar protein synthesis despite increases in FAK and anabolic signalling in L6 cells. J. Physiol. 587(Pt 14), 3719–3727. 10.1113/jphysiol.2009.169854 19470773 PMC2742293

[B7] Baar K. Torgan C. E. Kraus W. E. Esser K. (2000). Autocrine phosphorylation of p70(S6k) in response to acute stretch in myotubes. Mol. Cell Biol. Res. Commun. 4, 76–80. 10.1006/mcbr.2000.0257 11170836

[B8] Baccam A. Benoni-Sviercovich A. Rocchi M. Moresi V. Seelaender M. Li Z.. (2019). The mechanical stimulation of myotubes counteracts the effects of tumor-derived factors through the modulation of the activin/follistatin ratio. Front. Physiol. 10:401. 10.3389/fphys.2019.00401 31068826 PMC6491697

[B9] Bansai S. Morikura T. Onoe H. Miyata S. (2019). Effect of cyclic stretch on tissue maturation in myoblast-laden hydrogel fibers. Micromachines 10:399. 10.3390/mi10060399 31208059 PMC6630375

[B10] Bellott A. C. Patel K. C. Burkholder T. J. (2005). Reduction of caveolin-3 expression does not inhibit stretch-induced phosphorylation of ERK2 in skeletal muscle myotubes. J. Appl. Physiol. 98, 1554–1561. 10.1152/japplphysiol.01070.2004 15516368

[B11] Berggren J. R. Tanner C. J. Houmard J. A. (2007). Primary cell cultures in the study of human muscle metabolism. Exerc. Sport Sci. Rev. 35, 56–61. 10.1249/JES.0b013e31803eae63 17417051

[B12] Burkholder T. J. (2003). Permeability of C2C12 myotube membranes is influenced by stretch velocity. Biochem. Biophys. Res. Commun. 305, 266–270. 10.1016/S0006-291X(03)00756-3 12745068

[B13] Burkholder T. J. (2009). Stretch-induced ERK2 phosphorylation requires PLA2 activity in skeletal myotubes. Biochem. Biophys. Res. Commun. 386, 60–64. 10.1016/j.bbrc.2009.05.150 19524551 PMC2744880

[B14] Candiani G. Riboldi S. A. Sadr N. Lorenzoni S. Neuenschwander P. Montevecchi F. M.. (2010). Cyclic mechanical stimulation favors myosin heavy chain accumulation in engineered skeletal muscle constructs. J. Appl. Biomater. Biomech. 8, 68–75. 10.1177/228080001000800202 20740468

[B15] Carson J. A. Booth F. W. (1998). Effect of serum and mechanical stretch on skeletal alpha-actin gene regulation in cultured primary muscle cells. Am. J. Physiol. 275, C1438–C1448. 10.1152/ajpcell.1998.275.6.C1438 9843704

[B16] Chambers M. A. Moylan J. S. Smith J. D. Goodyear L. J. Reid M. B. (2009). Stretch-stimulated glucose uptake in skeletal muscle is mediated by reactive oxygen species and p38 MAP-kinase. J. Physiol. 587(Pt 13), 3363–3373. 10.1113/jphysiol.2008.165639 19403598 PMC2727043

[B17] Chromiak J. A. Vandenburgh H. H. (1992). Glucocorticoid-induced skeletal muscle atrophy *in vitro* is attenuated by mechanical stimulation. Am. J. Physiol. 262(6 Pt 1), C1471–C1477. 10.1152/ajpcell.1992.262.6.C1471 1616011

[B18] Chromiak J. A. Vandenburgh H. H. (1994). Mechanical stimulation of skeletal muscle cells mitigates glucocorticoid-induced decreases in prostaglandin production and prostaglandin synthase activity. J. Cell Physiol. 159, 407–414. 10.1002/jcp.1041590304 8188758

[B19] Clark C. B. Burkholder T. J. Frangos J. A. (2001). Uniaxial strain system to investigate strain rate regulation *in vitro*. Rev. Sci. Instrum. 72:2415. 10.1063/1.1362440

[B20] Clarke M. S. Feeback D. L. (1996). Mechanical load induces sarcoplasmic wounding and FGF release in differentiated human skeletal muscle cultures. FASEB J. 10, 502–509. 10.1096/fasebj.10.4.8647349 8647349

[B21] Collinsworth A. M. Torgan C. E. Nagda S. N. Rajalingam R. J. Kraus W. E. Truskey G. A. (2000). Orientation and length of mammalian skeletal myocytes in response to a unidirectional stretch. Cell Tissue Res. 302, 243–251. 10.1007/s004410000224 11131135

[B22] De Deyne P. G. (2000). Formation of sarcomeres in developing myotubes: role of mechanical stretch and contractile activation. Am. J. Physiol. Cell Physiol. 279, C1801–C1811. 10.1152/ajpcell.2000.279.6.C1801 11078695

[B23] Dewulf M. Koster D. V. Sinha B. C. Viaris de Lesegno Chambon V. Bigot A. Bensalah M.. (2019). Dystrophy-associated caveolin-3 mutations reveal that caveolae couple IL6/STAT3 signaling with mechanosensing in human muscle cells. Nat. Commun. 10:1974. 10.1038/s41467-019-09405-5 31036801 PMC6488599

[B24] Drummond M. J. Glynn E. L. Fry C. S. Timmerman K. L. Volpi E. Rasmussen B. B. (2010). An increase in essential amino acid availability upregulates amino acid transporter expression in human skeletal muscle. Am. J. Physiol. Endocrinol. Metab. 298, E1011–E1018. 10.1152/ajpendo.00690.2009 20304764 PMC2867366

[B25] Ebihara S. Hussain S. N. Danialou G. Cho W. K. Gottfried S. B. Petrof B. J. (2002). Mechanical ventilation protects against diaphragm injury in sepsis: interaction of oxidative and mechanical stresses. Am. J. Respir Crit. Care Med. 165, 221–228. 10.1164/ajrccm.165.2.2108041 11790659

[B26] Evans P. L. McMillin S. L. Weyrauch L. A. Witczak C. A. (2019). Regulation of skeletal muscle glucose transport and glucose metabolism by exercise training. Nutrients 11:2432. 10.3390/nu11102432 31614762 PMC6835691

[B27] Frenette J. Tidball J. G. (1998). Mechanical loading regulates expression of talin and its mRNA, which are concentrated at myotendinous junctions. Am. J. Physiol. 275, C818–C825. 10.1152/ajpcell.1998.275.3.C818 9730966

[B28] Gao S. Carson J. A. (2016). Lewis lung carcinoma regulation of mechanical stretch-induced protein synthesis in cultured myotubes. Am. J. Physiol. Cell Physiol. 310, C66-79. 10.1152/ajpcell.00052.2015 26491045 PMC4698448

[B29] Goto K. Okuyama R. Sugiyama H. Honda M. Kobayashi T. Uehara K.. (2003). Effects of heat stress and mechanical stretch on protein expression in cultured skeletal muscle cells. Pflugers Arch. 447, 247–253. 10.1007/s00424-003-1177-x 14534791

[B30] Guigni B. A. Fix D. K. Bivona J. J. 3rd Palmer B. M. Carson J. A. Toth M. J. (2019). Electrical stimulation prevents doxorubicin-induced atrophy and mitochondrial loss in cultured myotubes. Am. J. Physiol. Cell Physiol. 317, C1213–C1228. 10.1152/ajpcell.00148.2019 31532714 PMC6957384

[B31] Hanke N. Kubis H. P. Scheibe R. J. Berthold-Losleben M. Husing O. Meissner J. D.. (2010). Passive mechanical forces upregulate the fast myosin heavy chain IId/x via integrin and p38 MAP kinase activation in a primary muscle cell culture. Am. J. Physiol. Cell Physiol. 298, C910–C920. 10.1152/ajpcell.00265.2009 20071689

[B32] Hatfaludy S. Shansky J. Vandenburgh H. H. (1989). Metabolic alterations induced in cultured skeletal muscle by stretch-relaxation activity. Am. J. Physiol. 256(1 Pt 1), C175–C181. 10.1152/ajpcell.1989.256.1.C175 2912133

[B33] Hirata H. Sokabe M. Lim C. T. (2014). Molecular mechanisms underlying the force-dependent regulation of actin-to-ECM linkage at the focal adhesions. Prog. Mol. Biol. Transl. Sci. 126, 135–154. 10.1016/B978-0-12-394624-9.00006-3 25081617

[B34] Hornberger T. A. Armstrong D. D. Koh T. J. Burkholder T. J. Esser K. A. (2005). Intracellular signaling specificity in response to uniaxial vs. multiaxial stretch: implications for mechanotransduction. Am. J. Physiol. Cell Physiol. 288, C185–C194. 10.1152/ajpcell.00207.2004 15371259

[B35] Huang W. Liu X. Chen R. Feng L. Liao H. Yu L.. (2012). Effects of mechanical stretch with variant frequencies on alignment and differentiation of multilayer myotubes cultured *in vitro*. Zhongguo Xiu Fu Chong Jian Wai Ke Za Zhi 26, 735–742. 22792775

[B36] Hubatsch D. A. Jasmin B. J. (1997). Mechanical stimulation increases expression of acetylcholinesterase in cultured myotubes. Am. J. Physiol. 273, C2002–C2009. 10.1152/ajpcell.1997.273.6.C2002 9435507

[B37] Ilaiwy A. Quintana M. T. Bain J. R. Muehlbauer M. J. Brown D. I. Stansfield W. E.. (2016). Cessation of biomechanical stretch model of C2C12 cells models myocyte atrophy and anaplerotic changes in metabolism using non-targeted metabolomics analysis. Int. J. Biochem. Cell Biol. 79, 80–92. 10.1016/j.biocel.2016.08.012 27515590 PMC5067204

[B38] Iwata M. Hayakawa K. Murakami T. Naruse K. Kawakami K. Inoue-Miyazu M.. (2007). Uniaxial cyclic stretch-stimulated glucose transport is mediated by a ca-dependent mechanism in cultured skeletal muscle cells. Pathobiology 74, 159–168. 10.1159/000103375 17643061

[B39] Iwata M. Suzuki S. Hayakawa K. Inoue T. Naruse K. (2009). Uniaxial cyclic stretch increases glucose uptake into C2C12 myotubes through a signaling pathway independent of insulin-like growth factor I. Horm. Metab. Res. 41, 16–22. 10.1055/s-0028-1087170 18841528

[B40] Iwata Y. Katanosaka Y. Arai Y. Komamura K. Miyatake K. Shigekawa M. (2003). A novel mechanism of myocyte degeneration involving the Ca^2+^-permeable growth factor-regulated channel. J. Cell Biol. 161, 957-967. 10.1083/jcb.200301101 12796481 PMC2172975

[B41] Iwata Y. Katanosaka Y. Hisamitsu T. Wakabayashi S. (2007). Enhanced Na+/H+ exchange activity contributes to the pathogenesis of muscular dystrophy via involvement of P2 receptors. Am. J. Pathol. 171, 1576–1587. 10.2353/ajpath.2007.070452 17823278 PMC2043518

[B42] Johnston A. P. Baker J. De Lisio M. Parise G. (2011). Skeletal muscle myoblasts possess a stretch-responsive local angiotensin signalling system. J. Renin Angiotensin Aldosterone Syst. 12, 75–84. 10.1177/1470320310381795 20921089

[B43] Juffer P. Jaspers R. T. Klein-Nulend J. Bakker A. D. (2014). Mechanically loaded myotubes affect osteoclast formation. Calcif. Tissue Int. 94, 319-326. 10.1007/s00223-013-9813-8 24264813

[B44] Kim W. Kim J. Park H. S. Jeon J. S. (2018). Development of microfluidic stretch system for studying recovery of damaged skeletal muscle cells. Micromachines 9:671. 10.3390/mi9120671 30567359 PMC6315523

[B45] Kumar A. Xie L. Ta C. M. Hinton A. O. Gunasekar S. K. Minerath R. A.. (2020). SWELL1 regulates skeletal muscle cell size, intracellular signaling, adiposity and glucose metabolism. Elife 9:e58941. 10.7554/eLife.58941.sa2 32930093 PMC7541086

[B46] Lee W. J. Thompson R. W. McClung J. M. Carson J. A. (2003). Regulation of androgen receptor expression at the onset of functional overload in rat plantaris muscle. Am. J. Physiol. Regul. Integr. Comp. Physiol. 285, R1076–R1085. 10.1152/ajpregu.00202.2003 14557238

[B47] Liao I. C. Liu J. B. Bursac N. Leong K. W. (2008). Effect of electromechanical stimulation on the maturation of myotubes on aligned electrospun fibers. Cell. Mol. Bioeng. 1, 133–145. 10.1007/s12195-008-0021-y 19774099 PMC2747747

[B48] Lijnen P. Petrov V. (1999). Renin-angiotensin system, hypertrophy and gene expression in cardiac myocytes. J. Mol. Cell. Cardiol. 31, 949–970. 10.1006/jmcc.1999.0934 10336836

[B49] Lin S. S. Liu Y. W. (2019). Mechanical stretch induces mTOR recruitment and activation at the phosphatidic acid-enriched macropinosome in muscle cell. Front. Cell Dev. Biol. 7:78. 10.3389/fcell.2019.00078 31139627 PMC6518962

[B50] Massoulie J. Pezzementi L. Bon S. Krejci E. Vallette F. M. (1993). Molecular and cellular biology of cholinesterases. Prog. Neurobiol. 41, 31–91. 10.1016/0301-0082(93)90040-Y 8321908

[B51] McClung J. M. Lee W. J. Thompson R. W. Lowe L. L. Carson J. A. (2003). RhoA induction by functional overload and nandrolone decanoate administration in rat skeletal muscle. Pflugers Arch. 447, 345–355. 10.1007/s00424-003-1151-7 14556075

[B52] Mitsumoto Y. Downey G. P. Klip A. (1992). Stimulation of glucose transport in L6 muscle cells by long-term intermittent stretch-relaxation. FEBS Lett. 301, 94–98. 10.1016/0014-5793(92)80217-5 1451794

[B53] Mohammadkhah M. Murphy P. Simms C. K. (2016). The *in vitro* passive elastic response of chicken pectoralis muscle to applied tensile and compressive deformation. J. Mech. Behav. Biomed. Mater. 62, 468–480. 10.1016/j.jmbbm.2016.05.021 27281164

[B54] Moorwood C. Philippou A. Spinazzola J. Keyser B. Macarak E. J. Barton E. R. (2014). Absence of gamma-sarcoglycan alters the response of p70S6 kinase to mechanical perturbation in murine skeletal muscle. Skelet. Muscle 4:13. 10.1186/2044-5040-4-13 25024843 PMC4095884

[B55] Moustogiannis A. Philippou A. Zevolis E. Taso O. Chatzigeorgiou A. Koutsilieris M. (2020). Characterization of optimal strain, frequency and duration of mechanical loading on skeletal myotubes' biological responses. In Vivo. 34, 1779–1788. 10.21873/invivo.11972 32606147 PMC7439881

[B56] Murakami T. Yoshinaga M. (2013). Induction of amino acid transporters expression by endurance exercise in rat skeletal muscle. Biochem. Biophys. Res. Commun. 439, 449–452. 10.1016/j.bbrc.2013.08.094 24016666

[B57] Nakai N. Kawano F. Murakami T. Nakata K. Higashida K. (2018). Leucine supplementation after mechanical stimulation activates protein synthesis via L-type amino acid transporter 1 *in vitro*. J. Cell. Biochem. 119, 2094–2101. 10.1002/jcb.26371 28856713

[B58] Nakai N. Kawano F. Nakata K. (2015). Mechanical stretch activates mammalian target of rapamycin and AMP-activated protein kinase pathways in skeletal muscle cells. Mol. Cell. Biochem. 406, 285–292. 10.1007/s11010-015-2446-7 25971373

[B59] Nakamura T. Y. Iwata Y. Sampaolesi M. Hanada H. Saito N. Artman M.. (2001). Stretch-activated cation channels in skeletal muscle myotubes from sarcoglycan-deficient hamsters. Am. J. Physiol. Cell Physiol. 281, C690–C699. 10.1152/ajpcell.2001.281.2.C690 11443068

[B60] Nedachi T. Hatakeyama H. Kono T. Sato M. Kanzaki M. (2009). Characterization of contraction-inducible CXC chemokines and their roles in C2C12 myocytes. Am. J. Physiol. Endocrinol. Metab. 297, E866–E878. 10.1152/ajpendo.00104.2009 19622786

[B61] Nguyen H. X. Lusis A. J. Tidball J. G. (2005). Null mutation of myeloperoxidase in mice prevents mechanical activation of neutrophil lysis of muscle cell membranes *in vitro* and *in vivo*. J. Physiol. 565(Pt 2), 403–413. 10.1113/jphysiol.2005.085506 15790660 PMC1464517

[B62] Pardo P. S. Boriek A. M. (2012). An autoregulatory loop reverts the mechanosensitive Sirt1 induction by EGR1 in skeletal muscle cells. Aging 4, 456–461. 10.18632/aging.100470 22820707 PMC3433932

[B63] Pardo P. S. Mohamed J. S. Lopez M. A. Boriek A. M. (2011). Induction of Sirt1 by mechanical stretch of skeletal muscle through the early response factor EGR1 triggers an antioxidative response. J. Biol. Chem. 286, 2559–2566. 10.1074/jbc.M110.149153 20971845 PMC3024751

[B64] Perrone C. E. Fenwick-Smith D. Vandenburgh H. H. (1995). Collagen and stretch modulate autocrine secretion of insulin-like growth factor-1 and insulin-like growth factor binding proteins from differentiated skeletal muscle cells. J. Biol. Chem. 270, 2099–2106. 10.1074/jbc.270.5.2099 7530717

[B65] Peterson J. M. Pizza F. X. (2009). Cytokines derived from cultured skeletal muscle cells after mechanical strain promote neutrophil chemotaxis *in vitro*. J. Appl. Physiol. 106, 130–137. 10.1152/japplphysiol.90584.2008 18974369

[B66] Player D. J. Martin N. R. Passey S. L. Sharples A. P. Mudera V. Lewis M. P. (2014). Acute mechanical overload increases IGF-I and MMP-9 mRNA in 3D tissue-engineered skeletal muscle. Biotechnol. Lett. 36, 1113–1124. 10.1007/s10529-014-1464-y 24563297

[B67] Rauch C. Loughna P. T. (2005). Static stretch promotes MEF2A nuclear translocation and expression of neonatal myosin heavy chain in C2C12 myocytes in a calcineurin- and p38-dependent manner. Am. J. Physiol. Cell Physiol. 288, C593–C605. 10.1152/ajpcell.00346.2004 15483225

[B68] Rauch C. Loughna P. T. (2006). Cyclosporin-A inhibits stretch-induced changes in myosin heavy chain expression in C2C12 skeletal muscle cells. Cell Biochem. Funct. 24, 55–61. 10.1002/cbf.1187 15584088

[B69] Russell S. T. Sanders P. M. Tisdale M. J. (2006). Angiotensin II directly inhibits protein synthesis in murine myotubes. Cancer Lett. 231, 290–294. 10.1016/j.canlet.2005.02.007 16399230

[B70] Saito T. Okada S. Shimoda Y. Tagaya Y. Osaki A. Yamada E.. (2016). APPL1 promotes glucose uptake in response to mechanical stretch via the PKCzeta-non-muscle myosin IIa pathway in C2C12 myotubes. Cell. Signal. 28, 1694–1702. 10.1016/j.cellsig.2016.07.010 27478065

[B71] Sampaolesi M. Yoshida T. Iwata Y. Hanada H. Shigekawa M. (2001). Stretch-induced cell damage in sarcoglycan-deficient myotubes. Pflugers Arch. 442, 161–170. 10.1007/s004240100516 11417209

[B72] Sanders P. M. Russell S. T. Tisdale M. J. (2005). Angiotensin II directly induces muscle protein catabolism through the ubiquitin-proteasome proteolytic pathway and may play a role in cancer cachexia. Br. J. Cancer 93, 425–434. 10.1038/sj.bjc.6602725 16052213 PMC3217221

[B73] Sasai N. Agata N. Inoue-Miyazu M. Kawakami K. Kobayashi K. Sokabe M.. (2010). Involvement of PI3K/Akt/TOR pathway in stretch-induced hypertrophy of myotubes. Muscle Nerve 41, 100–106. 10.1002/mus.21473 19768770

[B74] Sinha B. Koster D. Ruez R. Gonnord P. Bastiani M. Abankwa D.. (2011). Cells respond to mechanical stress by rapid disassembly of caveolae. Cell 144, 402–413. 10.1016/j.cell.2010.12.031 21295700 PMC3042189

[B75] Soltow Q. A. Zeanah E. H. Lira V. A. Criswell D. S. (2013). Cessation of cyclic stretch induces atrophy of C2C12 myotubes. Biochem. Biophys. Res. Commun. 434, 316–321. 10.1016/j.bbrc.2013.03.048 23541574

[B76] Tidball J. G. Lavergne E. Lau K. S. Spencer M. J. Stull J. T. Wehling M. (1998). Mechanical loading regulates NOS expression and activity in developing and adult skeletal muscle. Am. J. Physiol. 275, C260–C266. 10.1152/ajpcell.1998.275.1.C260 9688857

[B77] Tidball J. G. Spencer M. J. Wehling M. Lavergne E. (1999). Nitric-oxide synthase is a mechanical signal transducer that modulates talin and vinculin expression. J. Biol. Chem. 274, 33155–33160. 10.1074/jbc.274.46.33155 10551887

[B78] Touyz R. M. Deng L. Y. He G. Wu X. H. Schiffrin E. L. (1999). Angiotensin II stimulates DNA and protein synthesis in vascular smooth muscle cells from human arteries: role of extracellular signal-regulated kinases. J. Hypertens. 17, 907–916. 10.1097/00004872-199917070-00006 10419063

[B79] Tsivitse S. K. Mylona E. Peterson J. M. Gunning W. T. Pizza F. X. (2005). Mechanical loading and injury induce human myotubes to release neutrophil chemoattractants. Am. J. Physiol. Cell Physiol. 288, C721–C729. 10.1152/ajpcell.00237.2004 15548571

[B80] Vandenburgh H. Kaufman S. (1979). *In vitro* model for stretch-induced hypertrophy of skeletal muscle. Science 203, 265–268. 10.1126/science.569901 569901

[B81] Vandenburgh H. Kaufman S. (1980). Protein degradation in embryonic skeletal muscle. Effect of medium, cell type, inhibitors, and passive stretch. J. Biol. Chem. 255, 5826–5833. 10.1016/S0021-9258(19)70703-0 7380836

[B82] Vandenburgh H. H. (1982). Dynamic mechanical orientation of skeletal myofibers *in vitro*. Dev Biol 93, 438–443. 10.1016/0012-1606(82)90131-2 7141107

[B83] Vandenburgh H. H. (1983). Cell shape and growth regulation in skeletal muscle: exogenous versus endogenous factors. J. Cell. Physiol. 116, 363–371. 10.1002/jcp.1041160314 6885933

[B84] Vandenburgh H. H. (1988). A computerized mechanical cell stimulator for tissue culture: effects on skeletal muscle organogenesis. In Vitro Cell. Dev. Biol. 24, 609–619. 10.1007/BF02623597 3397364

[B85] Vandenburgh H. H. Hatfaludy S. Karlisch P. Shansky J. (1989). Skeletal muscle growth is stimulated by intermittent stretch-relaxation in tissue culture. Am. J. Physiol. 256(3 Pt 1), C674–C682. 10.1152/ajpcell.1989.256.3.C674 2923199

[B86] Vandenburgh H. H. Hatfaludy S. Sohar I. Shansky J. (1990). Stretch-induced prostaglandins and protein turnover in cultured skeletal muscle. Am. J. Physiol. 259(2 Pt 1), C232–C240. 10.1152/ajpcell.1990.259.2.C232 2382700

[B87] Vandenburgh H. H. Karlisch P. (1989). Longitudinal growth of skeletal myotubes *in vitro* in a new horizontal mechanical cell stimulator. In Vitro Cell. Dev. Biol. 25, 607–616. 10.1007/BF02623630 2753848

[B88] Vandenburgh H. H. Kaufman S. (1981). Stretch-induced growth of skeletal myotubes correlates with activation of the sodium pump. J. Cell. Physiol. 109, 205–214. 10.1002/jcp.1041090203 7298728

[B89] Vandenburgh H. H. Kaufman S. (1982). Coupling of voltage-sensitive sodium channel activity to stretch-induced amino acid transport in skeletal muscle *in vitro*. J. Biol. Chem. 257, 13448–13454. 10.1016/S0021-9258(18)33469-0 6292191

[B90] Vandenburgh H. H. Shansky J. Karlisch P. Solerssi R. L. (1993). Mechanical stimulation of skeletal muscle generates lipid-related second messengers by phospholipase activation. J. Cell. Physiol. 155, 63–71. 10.1002/jcp.1041550109 8468370

[B91] Vandenburgh H. H. Shansky J. Solerssi R. Chromiak J. (1995). Mechanical stimulation of skeletal muscle increases prostaglandin F2 alpha production, cyclooxygenase activity, and cell growth by a pertussis toxin sensitive mechanism. J. Cell. Physiol. 163, 285–294. 10.1002/jcp.1041630209 7706373

[B92] Wang Y. Song J. Liu X. Liu J. Zhang Q. Yan X.. (2020). Multiple effects of mechanical stretch on myogenic progenitor cells. Stem Cells Dev. 29, 336–352. 10.1089/scd.2019.0286 31950873

[B93] Winter L. Staszewska I. Mihailovska E. Fischer I. Goldmann W. H. Schroder R.. (2014). Chemical chaperone ameliorates pathological protein aggregation in plectin-deficient muscle. J. Clin. Invest. 124, 1144–1157. 10.1172/JCI71919 24487589 PMC3934181

[B94] Wozniak A. C. Anderson J. E. (2009). The dynamics of the nitric oxide release-transient from stretched muscle cells. Int. J. Biochem. Cell Biol. 41, 625–631. 10.1016/j.biocel.2008.07.005 18694846

[B95] Xu X. Zhu F. Zhang M. Zeng D. Luo D. Liu G.. (2013). Stromal cell-derived factor-1 enhances wound healing through recruiting bone marrow-derived mesenchymal stem cells to the wound area and promoting neovascularization. Cells Tissues Organs 197, 103–113. 10.1159/000342921 23207453

[B96] Yamada M. Hokazono C. Tokizawa K. Marui S. Iwata M. Lira V. A.. (2019). Muscle-derived SDF-1alpha/CXCL12 modulates endothelial cell proliferation but not exercise training-induced angiogenesis. Am. J. Physiol. Regul. Integr. Comp. Physiol. 317, R770–R779. 10.1152/ajpregu.00155.2019 31577158

[B97] Yamashita-Goto K. Ohira Y. Okuyama R. Sugiyama H. Honda M. Sugiura T.. (2002). Heat stress facilitates stretch-induced hypertrophy of cultured muscle cells. J. Gravit. Physiol. 9, P145–P146. 15002522

[B98] Zhang J. S. Kraus W. E. Truskey G. A. (2004). Stretch-induced nitric oxide modulates mechanical properties of skeletal muscle cells. Am. J. Physiol. Cell Physiol. 287, C292–C299. 10.1152/ajpcell.00018.2004 15044149

